# Higher frequency of homologous chromosome pairing in adult endothelial cells as compared to in neonatal endothelial cells

**DOI:** 10.1242/jcs.264668

**Published:** 2026-10-23

**Authors:** Jemery Morales, Omar Akhtar, Gabriel Quintero Plancarte, Lisa Hua

**Affiliations:** Biology Department, Sonoma State University, Rohnert Park, CA 94928, USA

**Keywords:** Antipairing, Homologous chromosomes, Human aortic endothelial cells, Human umbilical vein endothelial cells, Mitosis

## Abstract

Homologous chromosomes are spatially segregated, or antipaired, in neonatal human endothelial cells at metaphase and anaphase, which might help prevent abnormal recombination. However, it is unclear whether this antipairing persists in adult endothelial cells. To test whether the antipairing, or one homolog per nuclear hemisphere motif, is conserved in adult endothelial cells, we examined human aortic endothelial cells at metaphase. Using immunolabeling with DNA fluorescent *in situ* hybridization (ImmunoFISH) and high-resolution confocal microscopy to visualize the chromosomes and centrosomes, we found that small homologous chromosomes 13, 15, 17, 19, 21, 22, and the sex chromosomes (XY) exhibit a loss of spatial segregation in human adult aortic endothelial cells. In addition, fewer adult endothelial cells showed segregation for the larger chromosomes 1, 4 and XX, as compared to in neonatal endothelial cells. Notably, we observed a higher frequency of abnormal pairing in both small and large chromosomes in adult aortic endothelial cells as compared to in neonatal umbilical vein endothelial cells. These findings suggest that mechanisms governing chromosome antipairing might decline with aortic endothelial cell age.

## Teaser

**Highlighted Article:** A higher frequency of homologous chromosome pairing is observed in human adult aortic endothelial cells than in neonatal umbilical vein endothelial cells.

## INTRODUCTION

Chromosomal organization is important for endothelial cell (EC) function, particularly during mitosis, when a cell divides to produce two identical daughter cells and maintain the correct chromosome number across cell divisions (Hua et al., 2022; Srinivasan et al., 2022). Non-random chromosome organization might play a role in maintaining genomic stability (Cai et al., 2025; Cremer and Cremer, 2001; Cremer et al., 2020; Hua and Mikawa, 2018b; Hua et al., 2022; Koeman et al., 2008; Meaburn et al., 2007; Misteli and Soutoglou, 2009). Research from our laboratory has shown that homologous chromosomes in neonatal human umbilical vein endothelial cells (HUVECs) are arranged on opposite sides of the centrosome axis during metaphase and early anaphase, resulting in a haploid set on either side (Cai et al., 2025; Hua and Mikawa, 2018b). This haploid set organization, or antipairing pattern, might minimize homologous chromosome pairing, indicating its importance in early development. However, it is unknown whether this antipairing pattern persists throughout adulthood.

Segregation of haploid genomes has also been observed in other model systems including the mouse, bovine, rabbit, fruit fly and nematode (Gondos and Bhiraleus, 1970; Hua et al., 2022; Joyce et al., 2012, 2016; Keneklis and Odartchenko, 1974; Mayer et al., 2000a,b; Maddox et al., 2005; Odartchenko and Keneklis, 1973; Reichmann et al., 2018; Schneider et al., 2021; Velez-Aguilera et al., 2020). This haploid set organization defines the earliest stages of development, with the observation of parental genome separation in mammalian models (Mayer et al., 2000a,b; Reichmann et al., 2018; Schneider et al., 2021). For example, in mouse and bovine cells, dual mitotic spindles at fertilization separate the two parental sets (Reichmann et al., 2018; Schneider et al., 2021). In addition, 5-bromodeoxyuridine (BrdU)-labeled sperm show paternal genome separation in mouse zygotes, which persist until the four-cell stage (Mayer et al., 2000b). Electron microscopy of fertilized rabbit ova shows intact parental pronuclei adjacent to each other until ∼21 h after mating (Gondos and Bhiraleus, 1970). After fertilization in the fruit fly, gonomeric cell divisions show the maternal and paternal genomes in apposition and structurally separate (Callaini and Riparbelli, 1996; Loppin et al., 2015). An evolutionary persistence might reflect selective pressure to limit harmful recombination and promote nuclear compartmentalization. In this study, we observe a postnatal degradation of the haploid set pattern in somatic mitotic cells.

Antipairing might be an important process in the fruit fly, as it can be inhibited or enhanced by different regulators, suggesting there are mechanisms driving the antipairing of homologous chromosomes in different animal models (Joyce et al., 2012, 2016). For example, in the early fruit fly embryo, a high-throughput FISH-based screen identifies genes involved in mitotic and interphase processes, promoting or antagonizing somatic homolog pairing (Joyce et al., 2012). This finding suggests that there may be active antipairing mechanisms to prevent the pairing of chromosomes in mammals. Taken together, these findings support the concept that antipairing is a biologically significant nuclear architecture strategy to maintain overall genomic stability.

Changes in chromatin structure further alter gene expression and increase susceptibility to DNA damage, with aging associated with higher levels of damage due to oxidative stress (Burgess et al., 2012; Wood and Helfand, 2013; Wood et al., 2010; Zhang et al., 2021). Notably, the spatial arrangement of chromosomes within the mitotic cell might become disorganized in aged ECs, which can impact gene expression and overall cellular function in interphase cells (Ghosh and Meyer, 2021; Heard and Bickmore, 2007; Hua et al., 2022; Koeman et al., 2008; Lima et al., 2022; Misteli, 2007). Aging disrupts nuclear organization through the loss of heterochromatin and nuclear lamina integrity, causing gene-rich regions to shift away from transcriptionally active domains and altering gene expression patterns (Chandra and Kirschner, 2016; Emerson and Lee, 2023; López-Otín et al., 2013).

The loss of antipairing might increase the chances of homologous chromosome pairing, leading to mitotic recombination and potential genomic instability. This loss has been correlated with renal cancer and altered gene expression (Hua and Mikawa, 2018b; Koeman et al., 2008). This finding suggests that abnormal somatic pairing and the loss of antipairing could be related to various human pathologies (Atkin and Jackson, 1996; Faruqi et al., 1994; Hua and Mikawa, 2018b; Hua et al., 2022; Koeman et al., 2008; Lewis et al., 1993). Despite this, there has been no thorough investigation into homologous chromosome organization in aged ECs, and a comprehensive analysis of this organization during mitosis is still lacking.

In this study, we employed 3D imaging to examine the organization of homologous chromosomes in adult human aortic endothelial cells (HAECs) sourced from both female and male individuals in the age range of 21 years to 68 years, and cultured at low passage numbers. Using immunofluorescence and DNA fluorescence *in situ* hybridization (ImmunoFISH) and high-resolution confocal microscopy, we analyzed the positions of homologous chromosomes to determine whether the spatial segregation of homologous chromosomes, or antipairing, persists in adult aortic ECs. Our findings reveal that mitotic antipairing is lost in adult aortic ECs. Small chromosomes 13, 15, 17, 19, 21, 22 and XY lost their spatial segregation in HAECs. In addition, fewer HAECs showed segregation as compared to in HUVECs for the larger chromosomes 1, 4 and XX. This suggests a gradual decline in the fidelity of spatial segregation of homologous and sex chromosomes in adult ECs. Both small and large chromosomes exhibited a higher frequency of abnormal homologous pairing in adult HAECs compared to that seen in neonatal HUVECs. The smaller chromosomes, including the acrocentric ones, showed a greater frequency of abnormal pairing than larger chromosomes in adult HAECs. There are at least two potential causes for this higher pairing frequency and the loss of antipairing – one would be an age-dependent change, and the other would be an aorta-specific mechanism to enhance the loss of antipairing. Overall, our data indicate that the mechanisms governing chromosome antipairing organization might decline, or lose fidelity, in aged aortic ECs.

## RESULTS

### Spatial segregation is maintained for small chromosomes 17 and 19 in neonatal HUVECs at metaphase

It was previously found that a pair of homologous chromosomes resides on opposite sides of the centrosome, or nuclear division axis, in human neonatal cells at metaphase and early anaphase (Hua and Mikawa, 2018b). This one homolog on either side of the centrosome axis, or antipairing, organization was conserved for all chromosomes in the human karyotype of HUVECs at early anaphase (Hua and Mikawa, 2018b). To confirm that antipairing is present at metaphase, individual chromosomes were visualized in HUVECs (Fig. 1A). HUVECs are polarized cells with an apical and basal domain (Muller and Gimbrone, 1986), which aided in the establishment of an axial coordinate system with a chromosome paint approach (Hua and Mikawa, 2018b). In addition, ImmunoFISH assays were performed to simultaneously visualize the centrosome axis (Fig. S1).

**Fig. 1.**
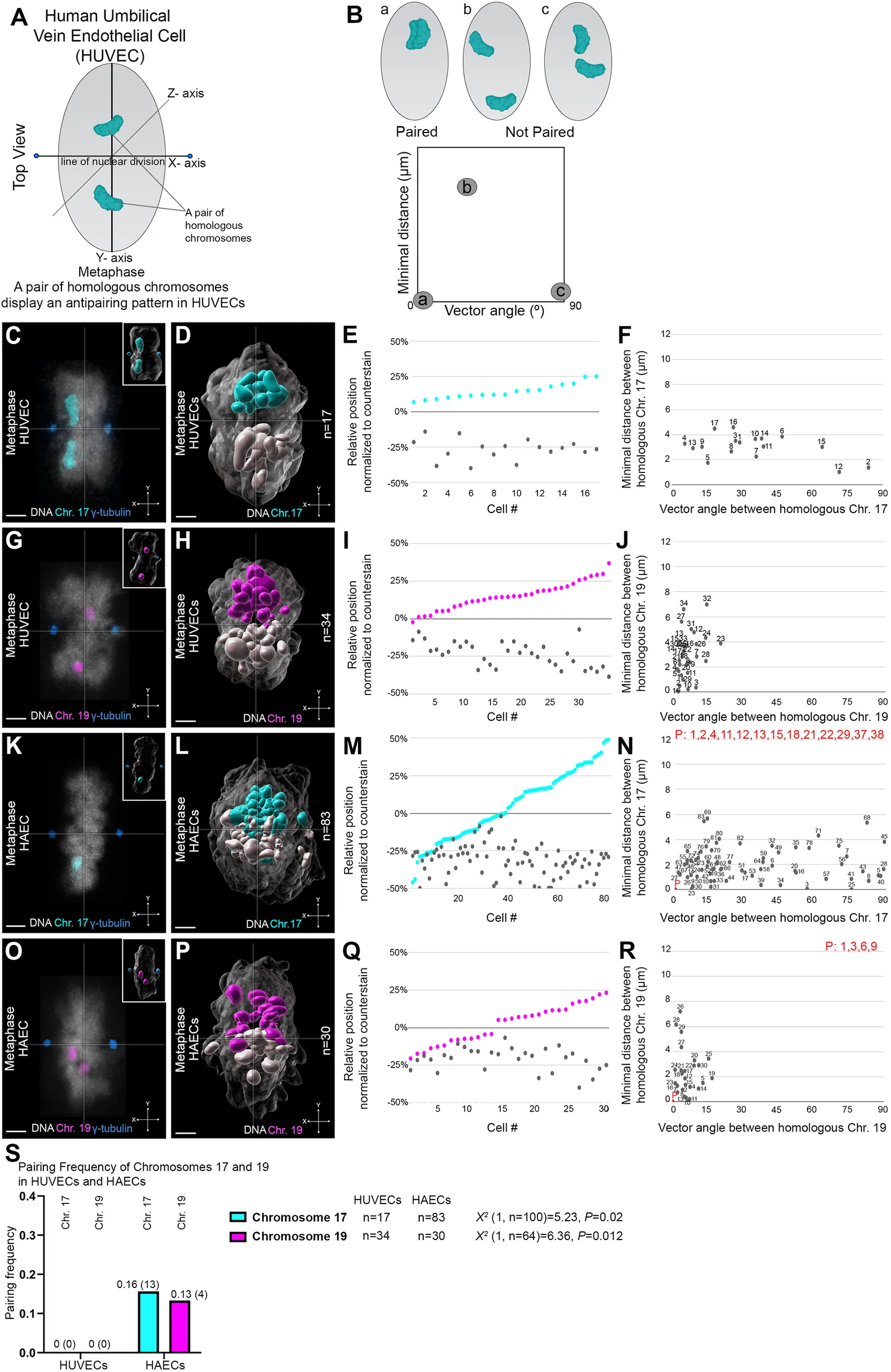
**Homologous chromosome segregation is lost in small chromosomes 17 and 19 in adult HAECs.** (A) Schematic showing a pair of homologous chromosomes localizing on opposite sides of the nuclear hemisphere for both autosomal and sex chromosomes in neonatal human umbilical vein endothelial cells (HUVECs) (Hua and Mikawa, 2018b). (B) Pairing is defined as the distance between two homologous chromosomes at 0 μm and vector angle at 0°. Different scenarios of paired (a) versus not paired (b, c) are shown. (C) Top view of optical sections of a HUVEC at metaphase stained for chromosomes 17 (cyan), γ-tubulin (blue) and counterstained for DNA (gray). Inset: 3D reconstruction of the optical sections. (D) A 3D overlay of chromosome 17 (cyan/gray) of multiple HUVECs at metaphase, where the homolog closest to the *x*-axis (*y*=0) is colored (cyan), whereas its respective partner is in gray (segregation in *n*=17/17 cells). (E) Relative positions for each homolog of chromosome 17 (cyan/gray circles) when mapped to an axial coordinate system. (F) The distance and angular orientation of homologous chromosome 17 pairs with an average distance of 3.03±0.99 μm and angular orientation of 34.5°±21.9° (mean±s.d.). Note: each numbered cell corresponds to the same cell number in the relative position normalized to the DNA counterstain. (G–J) As in C–F, but for chromosome 19 (segregation in *n*=33/34 cells). The cell in G,H is also shown in Figs S2 and S3A, and corresponds to cell #12 in Fig. S3Aʹ. The distance and angular orientation of chromosome 19 homologs with an average distance of 2.98±1.70 μm and angular orientation of 5.61°±4.27° (mean±s.d.). (K–N) As in C–F, but of adult human aortic endothelial cells (HAECs) (*n*=4 individuals, segregation in n=44/83 cells) with an average distance of 1.68±1.40 μm and angular orientation of 27.2°±27.5° (mean±s.d.). Note: red P indicates pairing and the following numbers are cells in which pairing has been identified. (O–R) As in G–J, but of HAECs. The cell in P is also shown in Fig. S3B. The distance and angular orientation of chromosome 19 homologs (n=4 individuals, segregation in *n*=17/30 cells) with an average distance of 1.99±1.89 μm and angular orientation of 5.43°±4.53° (mean±s.d.). (S) Pairing frequency graph showing the pairing of chromosomes 17 (cyan) χ^2^ (1, *n*=100)=5.23, *P*=0.02 and 19 (magenta) χ^2^ (1, *n*=64)=6.36, *P*=0.012 of HUVECs and HAECs. Pairing data were analyzed using binomial logistic regression. Numbers above the graph columns indicate pairing frequency and number of pairing events (given in brackets). Scale bars: 2 μm.

The nuclear hemispheres were defined by Hua and Mikawa (2018b) as the following: the *x*-axis is the line that crosses the centrosomes, the *z*-axis is the laser line, and the *y*-axis is perpendicular to both the *x*- and *z*-axes at metaphase and anaphase (Fig. 1A; Fig. S2A′–A‴). To evaluate the frequency of homologous pairing, we measured the minimal distance between each homologous pair (Hua and Mikawa, 2018b) (Fig. 1B). Pairing is defined as having a 0 μm distance and a 0° vector angle, determined in a 3D space, between a pair of homologous chromosomes (Fig. 1B). Thus, the spatial proximity of 0 μm distance and 0° vector angle is interpreted as direct spatial juxtaposition or contact along the axis of each homolog in our analysis (Fig. 1B). P and q arm orientation was not used to determine angular orientation of homologs due to the resolution of the chromosome paint probes that bind to sequences across the chromosome without distinction of p and q regions.

Our previous analysis of angular orientation was completed using a 180° range (Cai et al., 2025; Hua and Mikawa, 2018b). Angular orientation is determined by the angle between the 3D vectors along each homolog. Each vector starts at the point of origin of the centromere, and runs through the chromosome arm via the longest ellipsoid axis (Hua and Mikawa, 2018b). The vector angle was calculated by taking the inverse cosine of the dot product of two 3D vectors divided by the product of their magnitudes. At anaphase, spindle microtubules attach to kinetochores that surround centromeres of individual homologs that pull sister chromatids apart (Magidson et al., 2011; Walczak et al., 2010). This leads to chromosome arms trailing behind the centromeres (Magidson et al., 2011; Walczak et al., 2010).

There is less constraint of chromosome arms at metaphase and more dynamic movement (Cai et al., 2025; Magidson et al., 2011). At metaphase, the centromere position was less certain relative to the rest of the chromosome arm as centromeres or kinetochores oscillate parallel to the centrosome axis (Cai et al., 2025; Magidson et al., 2011). This stage difference makes it harder to identify the centromere position in fixed samples at metaphase, and does not allow us to assign directionality of vectors at the 180° range. Therefore, we limited our analysis to a single quadrant (90° range).

To determine whether the homologous chromosomes were segregated at metaphase, we performed ImmunoFISH to combine immunolabeling using an anti-γ-tubulin antibody to visualize the centrosomes (O'Toole et al., 2012), DNA FISH or whole chromosome paint (WCP), to visualize chromosomes, and a DNA counterstain (Fig. 1C,G,K,O). For WCP experiments, the centrosomes were not able to be visualized. Therefore, a DNA-derived axis perpendicular to the longest axis of the 3D metaphase DNA plate was used to determine the centrosome axis. Immunofluorescence staining for γ-tubulin, along with a DNA counterstain, revealed that the centrosome axis and DNA-derived axis had a difference of 0.5 μm (Fig. S1). Consequently, individual chromosome positions within ±0.5 μm of the DNA center of volume, referred to as the ‘boundary region’, were excluded from the analysis. For instance, out of 66 total cells, 6 cells exhibited homologous chromosome 1 in this boundary region, rendering the data uninterpretable (n=6/66 cells for chromosome 1).

We then determined whether a pair of homologous chromosomes resided on the same side or opposite side of the centrosome axis (Fig. S2A′–A‴). ImmunoFISH or WCP was carried out for samples of commercially available neonatal HUVECs (*n*=2 vials with 10 individuals pooled represented per vial, Table S1). Chromosomes 17 and 19 were selected based on differences in base pair number and chromosome structure pertaining to the p-arm (non-acrocentric) (Grimwood et al., 2004; Zody et al., 2006). In addition, chromosome 19 is a gene-dense chromosome that has been reported to exhibit abnormal homologous chromosome pairing in Caki-1 renal carcinoma cells and renal oncocytoma (Hua and Mikawa, 2018b; Koeman et al., 2008).

Metaphase cells were identified based on the highly condensed chromosomes aligned in the middle of the cell, with centrosomes positioned at the opposite poles (Walczak et al., 2010). Segregation of homologous chromosomes 17 and 19 was observed in a HUVEC at metaphase (Fig. 1C,G). To determine whether segregation of homologous chromosomes 17 and 19 occurred in multiple HUVECs, we created a 3D overlay (Hua and Mikawa, 2018b) (Fig. S2 for chromosome 19). The centrosome or *x*-axis, defined by positive γ-tubulin staining, or the center of DNA volume for each cell was used to establish an origin point (0,0,0) for alignment in the 3D overlay analysis (Fig. S2A′). The position of individual chromosomes was determined by identifying the center of volume of the segmented chromosome paint signal (Fig. S2A′). The 3D overlay of chromosomes 17 and 19 at metaphase demonstrated that multiple HUVECs displayed segregation of homologous chromosomes 17 and 19 along the centrosome axis (Fig. 1D,H; Fig. S2). Mapping of individual positions of the homologous chromosomes 17 and 19 in each HUVEC revealed that one homolog consistently positioned itself on each side of the centrosome axis (Fig. 1E,I; segregation in *n*=17/17 cells for chromosome 17, segregation in *n*=33/34 cells for chromosome 19; Fig. S3A,A′). In no case did we see homologous chromosomes 17 or 19 exhibit pairing at 0 μm distance or 0° angular orientation relative to its homolog similar to our previous study (Hua and Mikawa, 2018b) (Fig. 1F,J). These data demonstrated that chromosomes 17 and 19 were consistently antipaired in HUVECs at metaphase, and were organized in a non-random, antipaired configuration (Hua and Mikawa, 2018b).

### Homologous chromosome segregation is lost in small chromosomes 17 and 19 in adult HAECs

To investigate whether the antipairing pattern is present in adult aortic endothelial cells (HAECs), we performed ImmunoFISH or WCP for chromosomes 17 and 19 in single donor-derived HAECs (Table S1). Analyzing individual donor samples offer insights into whether this pattern is lost or conserved in adult aortic ECs across the general population. If the antipairing pattern is absent in multiple individuals, it might suggest that this loss is consistent across the adult aortic EC population. On the other hand, if the antipairing pattern is lost in some individuals but still present in others, it could indicate that the presence or absence is specific to individual people.

HAECs were sourced from individual deceased organ donor individuals of various ages, and purchased commercially (Lonza). Some donors had pathologies such as hypertension (*n*=2/7 individuals). For example, one of the 50-year-old females reported hypertension and glioblastoma, whereas the other 50-year old-female reported fibromyalgia and acute alcohol-related pancreatitis. The 68-year-old male reported hypertension, hyperlipidemia and diabetes. The other four remaining individuals did not report pathologies. In addition, not all individuals were analyzed for each chromosome.

To investigate whether the antipairing pattern was present across multiple individuals, we analyzed HAECs at metaphase from separate individuals (Table S1). For chromosome 17 analysis, HAECs from the 36-year-old female, both 50-year-old females and the 68-year-old male were analyzed (Table S1, *n*=4 individuals). For chromosome 19, HAECs from the 36-year-old female, 50-year-old male, one of the 50-year-old females and the 68-year-old male were analyzed (Table S1, *n*=4 individuals). Between chromosomes 17 and 19, HAECs were analyzed from five individuals in total (Table S1). We selected individuals from different age groups and sexes to determine whether the antipairing pattern was preserved among adult aortic ECs.

To determine whether small chromosomes maintain the spatial segregation of homologs in adult HAECs, we analyzed chromosomes 17 and 19 using ImmunoFISH/WCP. We found that both chromosomes 17 and 19 lose the pattern of one homolog per nuclear hemisphere in HAECs derived from five patients (Fig. 1K-R; segregation in=44/83 cells for chromosome 17, segregation in *n*=17/30 cells for chromosome 19; Fig. S3B,B′; Tables S1, S2). Quantification analysis revealed average distances of 1.68±1.40 μm and 1.99±1.89 μm, and angular orientations of 27.2°±27.5° and 5.43°±4.53° (all mean±s.d.) for homologous chromosomes 17 and 19, respectively (Fig. 1N,R; Tables S3, S4).

Notably, chromosome 17 was found to be abnormally paired in 13 out of 83 cells in three individuals, the 36-year-old and 50-year-old females and 68-year-old male (Fig. 1N; Table S1). The 50-year-old female and 68-year-old male also displayed pairing of chromosome 19 in four out of 30 cells (Fig. 1R). Together, these data show that small chromosomes 17 and 19 lose the spatial segregation and exhibit an increase of 15.0% abnormal pairing (17 out of 113 cells) in adult HAECs as compared to 0% of neonatal HUVECs (0 out of 51 cells) (Fig. 1S). A binomial logistic regression was performed to investigate the factors on the probability of pairing of chromosomes 17 and 19 in both HUVECs and HAECs [*χ*^2^ (1, n=164)=11.6, *P*<0.001].

However, both chromosomes 17 and 19 are small non-acrocentric chromosomes. To further investigate whether other small chromosomes that are acrocentric also lose the spatial segregation, we next mapped the position of small acrocentric chromosomes to determine whether the loss of spatial segregation is shared broadly among the human karyotype.

### Homologous segregation is lost in acrocentric chromosomes 13, 15, 21 and 22 in adult HAECs

Acrocentric chromosomes, including 13, 14, 15, 21, 22 and Y, are small chromosomes with the centromere located near one end of the chromosome, and contain the nucleolus-organizing region (Colaco and Modi, 2018; Henderson et al., 1972; Lejeune et al., 1960; Rogers, 2021). To test whether small acrocentric chromosomes also lose the one homolog per nuclear hemisphere pattern in adult HAECs, we selected chromosomes 13, 15, 21 and 22 to analyze.

The one homolog per nuclear hemisphere motif is a conserved pattern for acrocentric chromosomes in neonatal HUVECs (Hua and Mikawa, 2018b). Three-dimensional overlays of neonatal HUVECs showed segregation of chromosomes 13, 15, 21 and 22 at metaphase (Fig. 2A–L; Movie 1). Pairing of homologous chromosome 21 was observed in two out of 32 cells (Fig. 2I). These data confirm the homologous segregation of acrocentric chromosomes 13, 15, 21 and 22 in HUVECs, with almost no pairing.

**Fig. 2.**
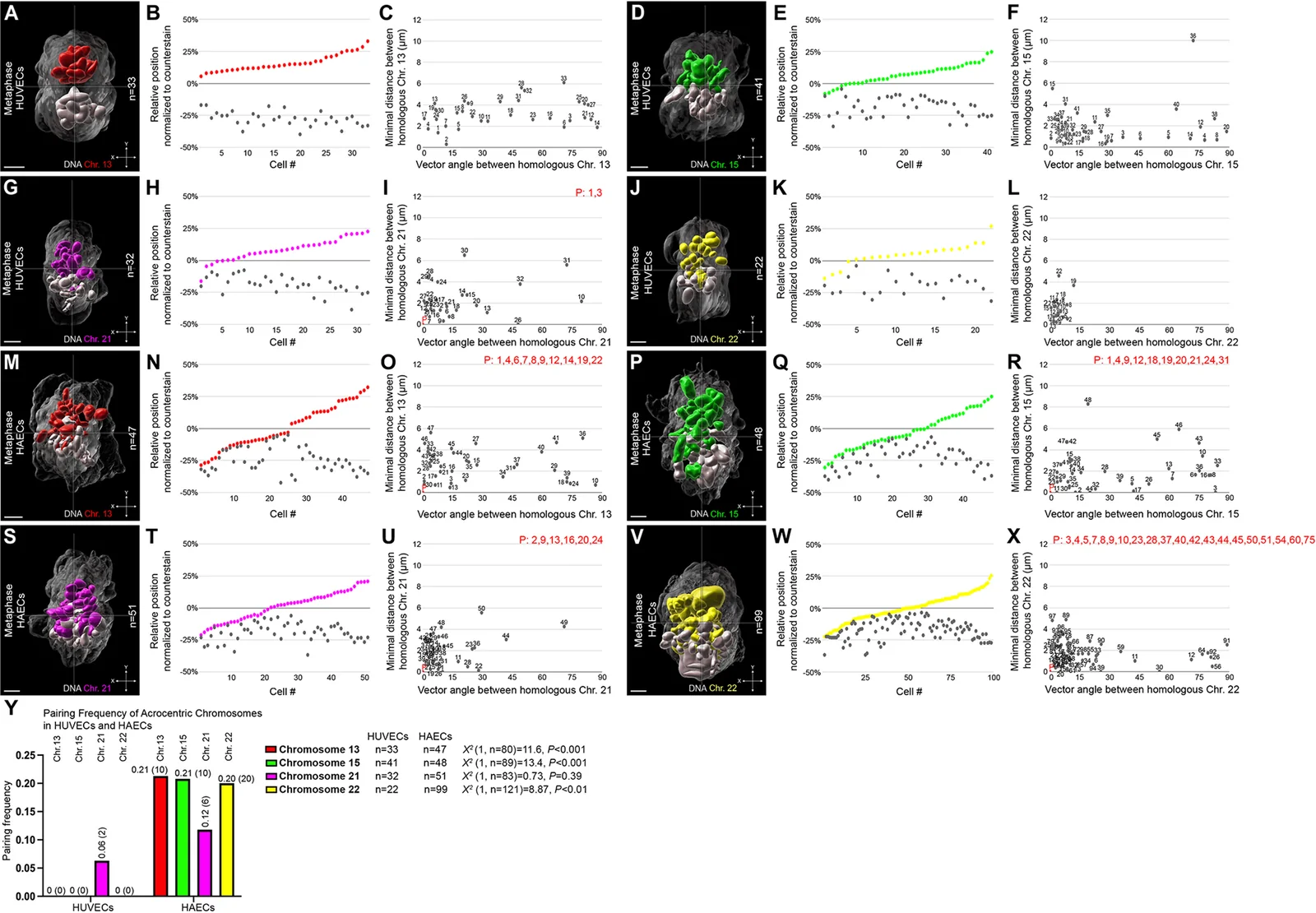
**Homologous chromosome segregation is lost in acrocentric chromosomes 13, 15, 21 and 22 in adult HAECs.** (A) A 3D overlay of chromosomes 13 (red/gray) of multiple neonatal HUVECs at metaphase (segregation in n*=*33/33 cells). (B) Relative positions for each homolog of chromosome 13 (red/gray circles) when mapped to an axial coordinate system. (C) The distance and angular orientation of homologous chromosome 13 pairs with an average distance of 3.19±1.27 μm and angular orientation of 39.7°±29.4° (mean±s.d.). Note: each cell is numbered as in Fig. 1*.* (D–F) As in A–C but for homologous chromosome 15 (green/gray) (segregation in *n*=33/41 cells). The distance and angular orientation of chromosome 15 homologs with an average distance of 1.88±1.72 μm and angular orientation of 27.5°±27.7° (mean±s.d.). (G–I) As in A–C but for chromosome 21 homologs (magenta/gray) (segregation in *n*=27/32 cells). The distance and angular orientation of chromosome 21 homologs with an average distance of 2.21±1.60 μm and angular orientation of 15.7°±20.3° (mean±s.d.). (J–L) As in A–C but for chromosome 22 homologs (yellow/gray) (segregation in *n*=18/22 cells). The distance and angular orientation of chromosome 22 homologs with an average distance of 1.50±1.09 μm and angular orientation of 4.56°±2.73° (mean±s.d.). (M–O) As in A–C, but of adult HAECs (n=4 individuals, segregation in *n*=22/47 cells) with an average distance of 2.06±1.66 μm and angular orientation of 20.8°±26.6° (mean±s.d.). (P–R) As in D–F, but of adult HAECs (*n*=3 individuals, segregation in *n*=19/48 cells). The distance and angular orientation of chromosome 15 homologs with an average distance of 1.67±1.85 μm and angular orientation of 26.6°±29.3° (mean±s.d.). (S–U) As in G–I, but of adult HAECs (*n*=4 individuals, segregation in *n*=30/51 cells) with an average distance of 1.81±1.35 μm and angular orientation of 8.78°±12.5° (mean±s.d.). (V–X) As in J–L, but of adult HAECs (n=7 individuals, segregation in *n*=48/99 cells). The distance and angular orientation of homologs of chromosome 22 with an average distance of 1.28±1.13 μm and angular orientation of 12.6°±20.0° (mean±s.d.). (Y) Pairing frequency graph showing the pairing of chromosomes 13 (red) χ^2^ (1, *n*=80)=11.6, *P*<0.001, 15 (green) χ^2^ (1, *n*=89)=13.4, *P*<0.001, 21 (magenta) χ^2^ (1, *n*=83)=0.73, *P*=0.39, 22 (yellow) χ^2^ (1, *n*=121)=8.87, *P*<0.01 of HUVECs and HAECs. Pairing data were analyzed using binomial logistic regression. Numbers above the graph columns indicate pairing frequency and number of pairing events (given in brackets). Scale bars: 2 μm.

In contrast, 3D overlays of adult HAECs for chromosomes 13, 15, 21 and 22 demonstrated a loss of the one homolog per nuclear hemisphere motif (Fig. 2M–X; Table S2). Abnormal pairing of the acrocentric chromosomes 13, 15, 21, and 22 was observed in 4 out of 4, 2 out of 3, 3 out of 4, and 3 out of 7 individuals, respectively (Fig. 2M–X; Table S1). Together, these data show that small acrocentric chromosomes 13, 15, 21 and 22 lose spatial segregation and exhibit a 18.8% increase of abnormal pairing (46 out of 245 cells) in adult HAECs as compared to 1.56% of neonatal HUVECs (2 out of 128 cells) [Fig. 2Y; Table S2; χ*^2^* (1, *n*=373)=30.7, *P*<0.0001]. The one homolog per nuclear hemisphere motif along the centrosome axis was lost for the acrocentric chromosomes 13, 15, 21 and 22 in seven adult individuals.

These data show that small chromosomes, both non-acrocentric and acrocentric, lose the one homolog per nuclear hemisphere motif and exhibit an increased frequency of homologous pairing. To rule out the possibility that a loss of antipairing and an increase in abnormal pairing events are not limited to small chromosomes only, we next investigated positioning of large chromosomes.

### Fewer adult HAECs show segregation for the larger chromosomes 1 and 4 as compared to in neonatal HUVECs

To test whether large chromosomes also lose the spatial segregation of homologous chromosomes, as observed in non-acrocentric and acrocentric adult HAECs, we mapped the positions of chromosomes 1 and 4 using ImmunoFISH or WCP. Compared to chromosomes 1 and 4 in HUVECs at metaphase, which showed consistent segregation along the centrosome axis (Fig. 3A–F), fewer HAECs displayed segregation of the larger chromosomes 1 and 4 (Fig. 3G–L) (*n*=5 individuals for each chromosome 1 and 4, respectively; Table S2). These findings indicate a gradual loss of fidelity in the spatial segregation of homologous chromosomes in adult aortic ECs.

**Fig. 3.**
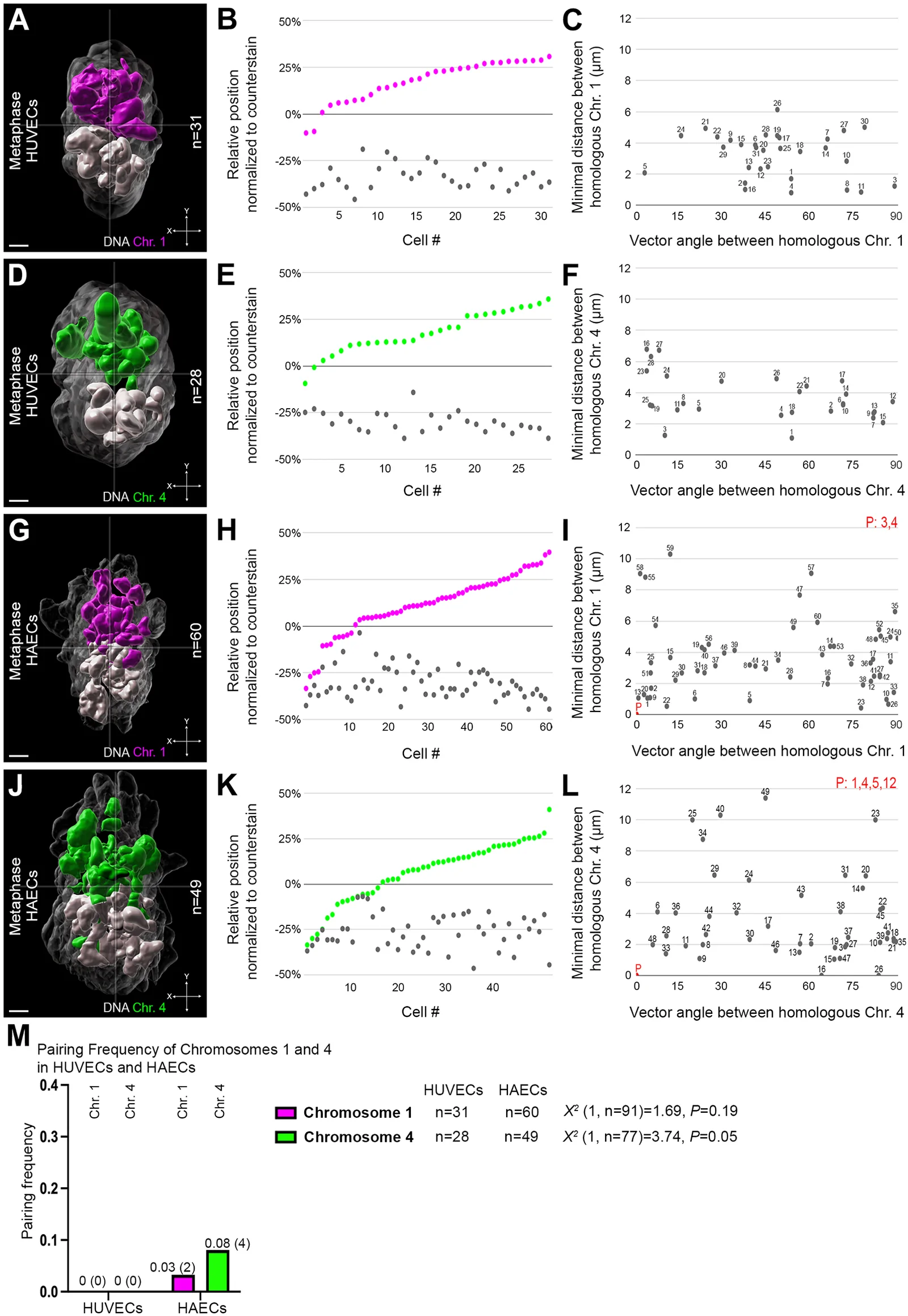
**Fewer adult HAECs show segregation for the larger chromosomes 1 and 4 as compared to what is seen in neonatal HUVECs.** (A) A 3D overlay of chromosome 1 (magenta/gray) of multiple neonatal HUVECs at metaphase (segregation in *n*=29/31 cells). (B) Relative positions for each homolog of chromosome 1 (magenta/gray circles) when mapped to an axial coordinate system. (C) The distance and angular orientation for the chromosome 1 homologs with an average distance of 3.25±1.45 μm and angular orientation of 48.2°±19.3° (mean±s.d.). Note: each cell is numbered as in Fig. 1*.* (D–F) As in A–C, but for chromosome 4 (segregation in *n*=26/28 cells). Chromosome 4 homologs have an average distance of 3.67±1.48 μm and angular orientation of 43.9°±30.8° (mean±s.d.). (G–I) As in A–C, but of adult HAECs. The distance and angular orientation chromosome 1 homologs (*n*=6 individuals, segregation in n=48/60 cells) with an average distance of 3.45±2.30 μm and angular orientation of 45.8°±32.3° (mean±s.d.). (J–L) As in D–F, but of adult HAECs (*n*=5 individuals, segregation in *n*=34/49 cells). The distance and angular orientation of chromosome 4 homologs with an average distance of 3.38±2.88 μm and angular orientation of 48.6°±30.1° (mean±s.d.). (M) Pairing frequency graph of chromosome 1 (magenta) χ^2^ (1, *n*=91)=1.69, *P*=0.19 and chromosome 4 (green) χ^2^ (1, *n*=77)=3.74, *P*=0.05 for both HUVECs and HAECs. Pairing data were analyzed using binomial logistic regression. Numbers above the graph columns indicate pairing frequency and number of pairing events (given in brackets). Scale bars: 2 μm.

Notably, pairing of homologous chromosomes 1 and 4 was observed in single HAECs for both the 21-year-old and 50-year-old males (Fig. 3I,L). Abnormal pairing of chromosomes 1 and 4 was observed in different cells. Two cells of the 50-year-old female also showed chromosome 4 pairing (Fig. 3L). In contrast, there was no abnormal pairing of chromosomes 1 and 4 in HUVECs (Fig. 3C,F). These data revealed that large chromosomes 1 (3.33%) and 4 (8.16%) in adult HAECs, with a combined 5.50% of cells (6 out of 109), displayed abnormal pairing. This contrasts with neonatal HUVECs, where no abnormal pairing was observed (0 out of 59 cells) [Fig. 3M; χ^2^ (1, *n*=168)=4.97, *P*=0.026]. Next, we investigated whether the sex chromosomes also exhibit a loss of spatial segregation and abnormal pairing in adult HAECs.

### Female adult HAECs show an increase in abnormal pairing of XX chromosomes as compared to XY chromosomes in male HAECs

To test whether the X chromosomes in females or the XY chromosomes in males lose their segregation pattern in adult HAECs, we performed ImmunoFISH or WCP (Fig. 4G–L). HUVECs have been reported to show spatial segregation of the XX and XY sex chromosomes along the centrosome axis in female and male neonatal HUVECs supporting that the mitotic antipairing organization is sex-type independent (Hua and Mikawa, 2018b). The XX and XY chromosomes in female and male HUVECs showed segregation (Fig. 4A–F). In no case did we observe homologous XX or XY chromosomes exhibit pairing at 0 μm distance or 0° angular orientation relative to its partner (Fig. 4C,F).

**Fig. 4.**
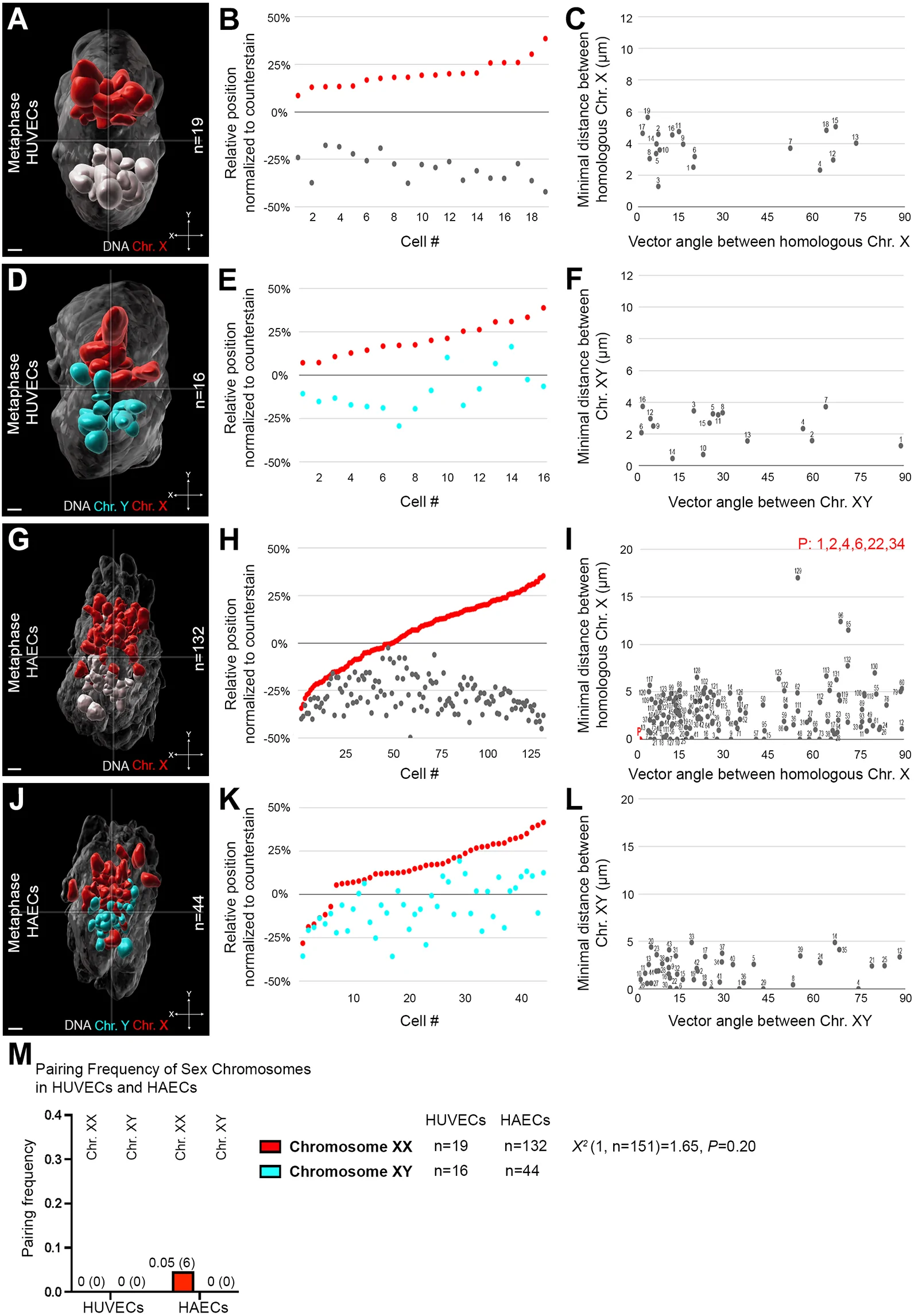
**Female adult HAECs showed an increase in abnormal pairing of XX chromosomes as compared to that in XY chromosomes in male HAECs.** (A) A 3D overlay of chromosome XX (red/gray) of multiple neonatal HUVECs at metaphase (segregation in *n*=19/19 cells). (B) Relative positions for each homolog of chromosome X (red/gray circles) when mapped to an axial coordinate system. (C) The distance and angular orientation of chromosome X homologs with an average distance of 3.79±1.08 μm and angular orientation of 26.5°±26.7° (mean±s.d.). Note: each cell is numbered as in Fig. 1. (D–F) As in A–C, but of XY (red/cyan) sex chromosomes (segregation in *n*=13/16 cells) with an average distance of 2.42±1.06 μm and angular orientation of 29.5°±25.5° (mean±s.d.). (G–I) Same as A–C, but of female-derived HAECs (*n*=4 individuals, segregation in *n*=82/132 cells). The distance and angular orientation of chromosome X homologs with an average distance of 2.79±2.57 μm and angular orientation of 35.2°±26.7° (mean±s.d.). (J–L) As in D–F, but of multiple male-derived HAECs (*n*=3 individuals, segregation in *n*=22/44 cells) with an average distance of 2.04±1.44 μm and angular orientation of 27.2°±24.6° (mean±s.d.). (M) Pairing frequency graph of chromosome XX (red), χ^2^ (1, *n*=151)=1.65, *P*=0.20, and chromosome XY (cyan) of HUVECs and HAECs. Pairing data were analyzed using binomial logistic regression. Note: statistical analysis could not be performed on chromosomes XY since no pairing was observed for both HUVECs and HAECs*.* Numbers above the graph columns indicate pairing frequency and number of pairing events (given in brackets). Scale bars: 2 μm.

However, female adult HAECs showed a loss of X chromosome segregation, and an increase in abnormal pairing as compared to that in HUVECs [Fig. 4G–I; Table S2; *χ*^2^ (1, *n*=151)=1.65, *P*=0.20]. Six cells from the 36-year-old, 45-year-old and 50-year-old female individuals showed abnormal pairing of 4.55% (*n*=6/132 cells). These data suggest that homologous chromosomes X, as well as the large chromosomes 1 and 4, exhibit similar abnormal pairing frequencies in adult HAECs.

Acrocentric autosomes in adult HAECs were shown above to be abnormally paired and lose their segregation (Fig. 2M–X). The Y chromosome is also an acrocentric chromosome (Colaco and Modi, 2018; Rogers, 2021). To test whether the XY chromosomes also lose the one sex chromosome per nuclear hemisphere motif in adult HAECs, we performed ImmunoFISH or WCP of three male donor-derived HAECs (Fig. 4J; Table S1). We found that XY chromosomes lose the one sex chromosome per hemisphere motif (Fig. 4J–L, *n*=44 cells; Table S2). Pairing of XY was not observed in contrast to the abnormal pairing observed in XX in female adult HAECs (Fig. 4I,L,M). These data suggest that there might be a greater affinity for chromosomes with similar sequence homology like the X chromosomes or homologous chromosomes to be abnormally paired as compared to heterologous XY chromosomes. Taken together, X chromosomes in HAECs showed an increased frequency of abnormal pairing as compared to XY chromosomes, with no pairing.

Collectively, small, large and the XX chromosomes showed a higher frequency of abnormal pairing in adult HAECs compared to neonatal HUVECs [Fig. 5A; χ^2^ (1, *n*=856)=42.5, *P*<0.0001].

**Fig. 5.**
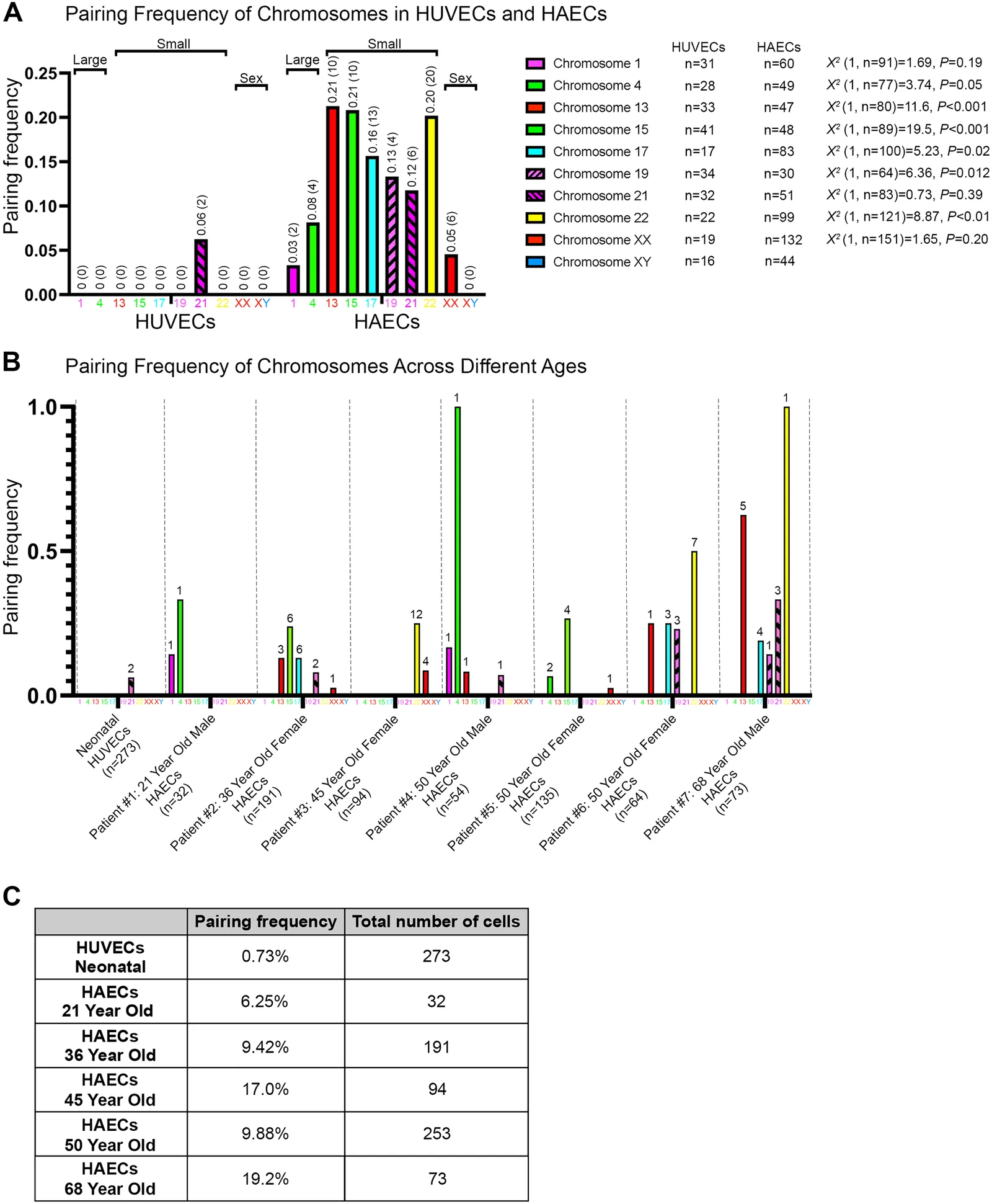
**Pairing frequency analysis showed smaller chromosomes had an increase in abnormal pairing as compared to larger chromosomes in adult HAECs.** (A) Chart representing pairing frequencies of HUVECs and HAECs across chromosomes 1 (magenta), 4 (green), 13 (red), 15 (green), 17 (cyan), 19 (magenta, fill pattern left), 21 (magenta, fill pattern right), 22 (yellow), XX (red) and XY (blue). The frequency of homologous chromosome pairing varies among the chromosomes analyzed [χ^2^ (9, *n*=916)=36.4, *P*<0.0001], for all chromosomes individually of HUVECs and HAECs {for chromosomes individually of HAECs [χ^2^ (9, n=643)=39.2, *P*<0.0001]}. For all chromosomes based on sizing of small, large, and sex chromosomes of HUVECs and HAECs [χ^2^ (2, *n*=916)=26.1, *P*<0.0001]. Pairing data were analyzed using binomial logistic regression. Numbers above the graph columns indicate pairing frequency and number of pairing events (given in brackets). (B) Chart of pairing frequency of chromosomes across different age groups. Note: numbers above bars indicate the number of pairing events for each chromosome. (C) Table of pairing frequency percentage and sample sizes of analyzed cells across different age groups.

### Pairing frequency analysis shows smaller autosomes have an increase in abnormal pairing as compared to what is seen for larger chromosomes and sex chromosomes in adult HAECs

It has been proposed that the loss of the antipairing organization can increase the likelihood of somatic homologous chromosome pairing and mitotic recombination, due to the close proximity of the homologs (Hua and Mikawa, 2018b). The loss of segregation leads to an increased frequency of abnormal pairing observed for chromosomes 1, 4, 13, 15, 17, 19, 21, 22 and XX, with the exception of XY sex chromosomes, in adult HAECs (Fig. 5A). Although fewer HAECs within the HAEC samples showed a loss of spatial segregation of the large chromosomes 1, 4 and XX in females, this loss was still detectable (Table S2).

Smaller autosomes were observed to have an increase in abnormal pairing as compared to what was seen for larger autosomes and sex chromosomes in HAECs [*χ*^2^ (2, *n*=643)=31.3, *P*<0.0001]. For example, chromosomes 1, 4 and XX, which are larger, showed less pairing (4.98%, 12 out of 241 cells), as compared to that seen for smaller chromosomes 13, 15, 17, 19, 21 and 22, which exhibited increased abnormal pairing (17.6%, 63 out of 358 cells) (Fig. 5A). In addition, acrocentric chromosomes compared to non-acrocentrics also had an increased frequency of pairing events [*χ* ^2^ (1, *n*=643)=9.18, *P*=0.0024]. Our data suggest the underlying mechanisms of homologous segregation and pairing differentially impact chromosomes of different sizes of HAECs (Table S2).

## DISCUSSION

The spatial separation of homologous chromosomes along the centrosome axis, or antipairing, likely plays a fundamental role in maintaining nuclear organization and genome stability. Beyond preventing mitotic recombination, antipairing might serve several crucial functions, including preserving nuclear architecture, minimizing inappropriate interchromosomal interactions, ensuring the fidelity of chromosome segregation during mitosis and supporting precise gene expression programs. Disruption of this organization could therefore have significant consequences for cellular function and genomic integrity.

Previous work has shown that neonatal ECs display spatial segregation of homologous chromosomes during metaphase and early anaphase (Cai et al., 2025; Hua and Mikawa, 2018b). Here, we find that in adult aortic ECs, the spatial segregation of homologous chromosomes is lost for small chromosomes and the XY sex chromosomes. Notably, segregation and pairing frequencies vary by chromosome size in HAECs, with smaller chromosomes showing a loss of segregation, and a higher frequency of pairing compared to what occurs for large chromosomes (Fig. 5A; Table S2). The loss of spatial segregation leads to an increased frequency of pairing of chromosomes 13, 15, 17, 19, 21 and 22, with the exception of XY sex chromosomes, in HAECs (Fig. 5A; Table S2). Although fewer adult HAECs showed a loss of segregation of chromosomes 1, 4 and XX, there was an increased frequency of pairing observed when compared to HUVECs (Fig. 5A; Table S2).

These observations raise the possibility that the loss of antipairing is not uniform across the genome but might follow structural or positional rules. This differential homolog pairing based on chromosome number and morphology provides mechanistic hints underlying the antipairing organization. These findings suggest that after birth there might be an increased susceptibility to a loss of nuclear organization, in particular impacting small acrocentric chromosomes (Fig. 5B,C).

Consistent with our observations of homologous segregation, prior studies using fixed HeLa cell and human fibroblast analysis at mitosis show a nonrandom, antiparallel arrangement of homologous chromosomes (Nagele et al., 1995, 1998). Although we did not find an antiparallel configuration of homologous chromosomes (Hua and Mikawa, 2018b), these prior publications support that a nonrandom chromosome organization pattern, such as haploid set segregation, is present.

Live-imaging analysis of hTERT-RPE-1 cells report a random distribution of homologous chromosomes (Stamatov et al., 2025). One interpretation is that immortalized cell lines might not display the same antipairing organization as in primary human cells because antipairing mechanisms might not be present.

In the small chromosomes that were analyzed, we found that the acrocentric chromosomes 13, 15 and 22 had a higher pairing frequency than the small non-acrocentric chromosomes 17 and 19 (Fig. 5A). One possible explanation for the differential behavior of these acrocentric versus non-acrocentric chromosomes is the presence of interchromosomal contacts among acrocentrics (Henderson et al., 1973; Potapova et al., 2019). These chromosomes, which are involved in nucleolus assembly, also contain interchromosomal DNA connections (Lezhava, 1984; Potapova et al., 2019). It is plausible that these interchromosomal connections are disrupted first, destabilizing acrocentric chromosomes and eventually influencing broader chromosomal organization. However, acrocentric chromosome 21 had a lower pairing frequency (of 0.12) than other acrocentric chromosomes in HAECs (Fig. 5A). Chromosome 21 also displayed two pairing events in HUVECs, suggesting this acrocentric chromosome might behave differently to others (Fig. 5A).

Mitotic recombination events between heterologous acrocentrics have also been reported (Guarracino et al., 2023; Guissani et al., 1996). Moreover, smaller chromosomes tend to localize more centrally within the metaphase plate, along the centrosome axis, than larger chromosomes, suggesting a size-dependent spatial organization (Hua and Mikawa, 2018b; Hua et al., 2022; Sun et al., 2000).

Research has highlighted a link between chromosome organization and aging (Chandra and Kirschner, 2016; Guarente, 1996; Lezhava, 2001; Liu et al., 2022; Naylor and van Deursen, 2016). As ECs age, they exhibit decreased proliferative capacity, leading to senescence, impaired wound healing and a decline in vascular repair (Bhayadia et al., 2016; Bloom et al., 2023a,b; Guo et al., 2022; Jia et al., 2019; Liu et al., 2019; Rippe et al., 2012; Shabanian et al., 2024; Ting et al., 2021). One common feature is aneuploidy, an abnormal number of chromosomes that can impair normal cell function, which is associated with vascular aging (Donato et al., 2015, 2018). For example, human aortic ECs derived from individuals 30 years and older show a higher frequency of aneuploidy (such as tetrasomy) compared to ECs from younger donors (18 months to 30 years old) (Aviv et al., 2001).

Small chromosomes, in particular chromosome 13, have been reported to be gained or lost with age, and upon passaging of ECs and in hematological disorders (Aviv et al., 2001; Hamuro et al., 2016; Kimura et al., 2004; Miyai et al., 2008; Zhang et al., 2000). This pattern of instability might also extend to the sex chromosomes (Guttenbach et al., 1995; Hando et al., 1994; Martin et al., 1980; Miyai et al., 2008; Nowinski et al., 1990; Richard et al., 1994; Stone and Sandberg, 1995). The Y chromosome has been shown to exhibit age-related loss in several tissues (Aviv et al., 2001; Catalán et al., 1998; Gutiérrez-Hurtado et al., 2024; Nath et al., 1995; Pierre and Hoagland, 1972; Sakurai and Sandberg, 1976; Wiktor et al., 2000; Zhang et al., 2007). Although the XY sex chromosomes lose the segregation pattern, they do not show increased pairing events like the autosomes (Fig. 4J–L, Fig. 5A; Table S2). This difference likely reflects their unique sequence properties and structural organization. The XY chromosomes share only limited sequence homology, primarily in the pseudoautosomal regions (PAR1 and PAR2 regions) (Helena Mangs and Morris, 2007), and undergo independent pairing mechanisms distinct from those of homologous autosomes in both developmental and aging processes (Gutiérrez-Hurtado et al., 2024; Jacobs et al., 2012; Nath et al., 1995; Weng et al., 2016). Consistent with this, the Y chromosome also undergoes frequent loss in aging tissues, particularly in the hematopoietic and blood cell lineages (Aviv et al., 2001; Gutiérrez-Hurtado et al., 2024; Jacobs et al., 1963; Miyai et al., 2008; Nath et al., 1995; Pierre and Hoagland, 1972; Sakurai and Sandberg, 1976; Stone and Sandberg, 1995; Weng et al., 2016; Wiktor et al., 2000; Zhang et al., 2007). It might be uniquely vulnerable to genomic instability due to its structure, limited recombination and small size (Colaco and Modi, 2018; Rogers, 2021).

Owing to the challenges in culturing human adult aortic ECs, we were only able to analyze samples from seven adult organ donor individuals: one male each aged 21, 50, and 68, two females aged 36 and 45, and two females aged 50 (Table S1). As a result, we cannot exclude the possibility that these individual cases might not reflect the broader population for chromosome organization (Lau et al., 2020).

We observed abnormal pairing of homologous chromosomes for all seven individuals, rather than only a few. This suggests the loss of antipairing is a common feature in adult aortic ECs (Fig. 5C). Whether the loss of antipairing in adult HAECs increases with age and/or is associated with sex still remains elusive. Our study analyzed a subset of individuals across different age groups, which limited statistical analysis of age, and age with sex determinants (Table S1). Nonetheless, graphical data suggests a preliminary age-based loss of spatial segregation of homologous chromosomes, with a trend in the increase of pairing frequency during adulthood (Fig. 5B).

There are at least two potential causes for the higher pairing frequency or loss of antipairing: one could be an age-dependent change, whereas the other might involve an aorta-specific mechanism that enhances the loss of antipairing. These hypotheses, age-related versus tissue-specific, can be independently evaluated.

Importantly, the spatial arrangement of chromosomes within the cell can become disordered in adult aortic ECs, potentially affecting gene expression and overall cellular function. Our findings offer a better understanding of the mitotic antipairing pattern observed in human neonatal ECs, which may be lost with age. This loss of higher-order chromosome organization highlights its crucial role in maintaining genomic stability in human cells.

## MATERIALS AND METHODS

### Cell culture

Primary human umbilical vein endothelial cells (HUVECs, ATCC, Manassas, VA, USA; cat. no. PSC-100-013, Lot# 70032758 and 81006213) were cultured according to established protocols (Cai et al., 2025; Hua and Mikawa, 2018b). Each lot consisted of pooled neonatal HUVECs from ten donors, representing a total of 20 individual donor samples across both lots. The study did not involve collection from human participants. Cell lines in the study were purchased through commercial vendors (ATCC, and Lonza).

Human aortic endothelial cells (HAECs), (Lonza, Basel, Switzerland; cat. no. CC-2535, Lot# 0000430739, 22TL13791, 000225231, 0000351486, 0000337673, 21TL076036 and 22TL073027) were derived from individual human donors (21 year old male, 36 year old female, 45 year old female, 50 year old male, two 50 year old females, and 68 year old male) and were fixed at low passage (under seven). MCDB-131 (Gibco, cat no. 10372-019), 1% Glutamax (Gibco, cat. no. 35050-061), 1% penicillin-streptomycin (Gibco, cat. no. 15140-122), and 2% large vessel endothelial supplement (Gibco, cat. no. A14608-01) were used for cell growth. Cells were grown on a 10 cm dish until reaching a confluency of 70–80% and then transferred onto a 15 cm plate containing two-well, 8 mm well diameter PTFE glass slides [Electron Microscopy Sciences (Hatfield, PA, USA; cat. no. 63416-08)], which were flamed or UV sterilized.

### Immunofluorescence and chromosome painting

HUVECs and HAECs were grown on PTFE glass slides to 80–90% confluence. HUVECs and HAECs were not synchronized prior to metaphase analysis to minimize potential confounding effects associated with cell synchronization procedures (Rodman et al., 1980; Rohlf et al., 1980). Cells were fixed with 4% paraformaldehyde to preserve the three-dimensional nuclear and chromosomal architecture as paraformaldehyde fixation is widely recognized for maintaining nuclear integrity (Hepperger et al., 2007; Hobro and Smith, 2017; Hua and Mikawa, 2018a,b; Kozubek et al., 2000). Following fixation, the slides were stored in 70% ethanol at −20°C for a minimum of 24 h. Imaging was performed immediately after completion of the experimental procedures, following curing of the samples in ProLong Antifade mounting medium, according to manufacturer's instructions.

### Immunofluorescence

Slides were washed three times in 4°C 1× phosphate-buffered saline (PBS) on ice before undergoing heat-induced antigen retrieval for 10 min in a sodium citrate buffer solution (10 mM sodium citrate, 0.05% Tween-20, pH 6.0) in a steamer. Slides were then washed in a permeabilization buffer (0.25% Triton X-100 in 1× PBS) for 10 min followed by three 1× PBS washes at room temperature (RT). A blocking buffer (10% goat serum, 0.1% Tween-20 in 1× PBS) was then applied for 30 min at RT. Slides were incubated with a primary antibody to γ-tubulin [1:1000, rabbit anti-γ-tubulin antibody (Abcam, Cambridge, UK), cat. no. ab11317, RRID:AB_297921] diluted in 10% goat serum in 1× PBS with 0.1% Tween-20. Parafilm was used to cover slides. Slides were then incubated at 4°C in a humidified chamber overnight. The following day, the slides were washed three times with 1× PBS. Incubation of a secondary antibody (1:500, abcam: Goat Anti-Rabbit IgG H&L (Alexa Fluor^®^ 647) (Abcam cat. no. ab150079, RRID:AB_2722623) (in 1% BSA in 1× PBS with 0.1% Tween-20) was applied to slides, covered in Parafilm, and incubated in the dark for 1 h at RT. Slides were then washed twice with 1× PBS, and processed with DNA counterstain DAPI (Invitrogen: D1306) for immunofluorescence. The slides were washed twice in 1× PBS. After, Antifade (Invitrogen, cat. no. P36930) was applied with a 24×60 mm coverslip (Corning, NY, USA, cat. no. 2980-246, #1.5) and sealed with nail polish. The slides were stored at 4°C until using the confocal microscope.

### Immunofluorescence and chromosome painting

If continuing on for chromosome painting, before processing with the DNA counterstain steps listed above, the slides were incubated with an EGS crosslinker solution [25% DMSO, 0.375% Tween-20, 25 mM EGS (Thermo Scientific, cat. no. 21565) in 1× PBS] for 10 min in the dark at RT. Slides were washed twice with 1× PBS. WCPs for chromosomes 1, 4, 13, 15, 17, 19, 21, 22, X and Y in Aqua, Texas Red or FITC [Applied Spectral Imaging (ASI), Carlsbad, CA, USA] were used to visualize individual chromosomes in metaphase cells.

Chromosome paint probes (ASI) are generated using flow-sorted chromosomes. Degenerate oligonucleotide-primed (DOP)-PCR is used for DNA amplification with a semi-degenerate primer that anneals at random positions across the genome, with a bias towards primer binding site regions with more compatible 6-mer sequences. Thousands of DNA fragments are generated for each chromosome probe, ranging in size from ∼200 bp to ∼2000 bp, with a peak distribution at ∼400–800 bp. The coverage of these fragments is broad but not uniform, with an estimation of ∼70–85% coverage of the whole chromosome and similar for all chromosomes. Repetitive sequences such as centromeres and telomeres are underrepresented as they are blocked by Cot-1 DNA. The rDNA and X/Y homologous sequences can also display cross-hybridization artifacts and non-specific signals on the acrocentric and sex chromosomes.

The probes were preheated on an 80°C heat block for 10 min. Then, the probes were incubated at 37°C for 1 h. The slides were washed with a buffer containing saline-sodium citrate (2× SSC, Invitrogen, cat. no. 15557-044). After, the slides were submerged in 0.1 M HCl for 5 min at RT. Then, the slides were washed with 1× PBS, three times for 5 min, followed by a series of chilled ethanol washes on ice. The slides were placed in a denaturation solution (20× SSC, formamide, deionized water) for 7 min at 80°C followed by a second series of cold ethanol washes on ice. The slides were incubated with probes on a slide warmer. An 8 mm (World Precision Instruments, Sarasota, FL, USA, cat. no. 052041) and 12 mm coverslip (Electron Microscopy Sciences, cat. no. 72290-04) were stacked in subsequent order on the wells, and sealed with rubber cement. The slides were incubated in a moisturizing container containing formamide and deionized water, overnight at 37°C. The following day, the slides were washed in a post-hybridization buffer (1% Tween-20, 10% 20× SSC in deionized water) at 42°C for 10 min, three times. Slides were then incubated with a DNA counterstain (DAPI, Hoechst 33342, SYTOX, or TO-PRO-3, Invitrogen; cat. no. D1306, H1399, S11380 and T3605). The slides were washed twice in 1× PBS. After, Antifade was applied with a 12 mm coverslip and sealed with nail polish. The slides were stored at 4°C.

### Confocal microscopy

Fixed cells were imaged using a laser scanning confocal microscope (Leica, Wetzlar, Germany, TCS SPE, DM2500) using a 63×/1.3NA oil immersion objective with a digital zoom of 1.5× to visualize individual cells. The *z*-stacks (*z*-step size 0.35 μm) were acquired sequentially in a four-channel mode; *z*-stacks were captured using a frame size of 1024×1024 pixels and processed with Leica Application Suite X software (version: 3.5.2.18963).

### 3D reconstruction and overlay of homologous chromosomes

Confocal optical sections were reconstructed in a 3D software, Imaris (Bitplane, 9.7.2). Three-dimensional overlay generation of homologous chromosomes in multiple fixed metaphase HUVECs and HAECs was performed as previously described (Hua and Mikawa, 2018b). We detected a small population of HAECs to be polyploid, consistent with prior reports (Aviv et al., 2001; Borradaile and Pickering, 2010). For example, we found 36 of ∼207,000 HAECs to be polyploid, and these cells were not included in the analysis.

To distinguish homolog pairing from chromosome loss (monosomy), we quantified chromosome paint signal volume. The volume of a paired chromosome paint signal was approximately equivalent to the combined volume of two individual homologs. For example, the average volume of an individual, antipaired, chromosome 19 homolog paint signal was measured to be 2.15±1.02 μm^3^ in 10 HUVECs and 2.32±1.08 μm^3^ in 10 HAECs (mean±s.d.). However, the average volume of the single chromosome paint signals, in the four cases of chromosome 19 pairing in HAECs, was 5.12±3.38 μm^3^. These average volumes showed that the paired chromosome 19 paint signals in HAECs were approximately twice the volume of the individual antipaired homologous chromosome 19 paint signals in HUVECs and HAECs, respectively.

Multiple pairs of homologous chromosomes were visualized by creating the cellular axis that was defined by Hua and Mikawa (2018b). The *x*, *y* and *z* axes were set an origin of 0,0,0 to define each nuclear hemisphere across the *x*-axis (centrosome axis), and to analyze how each pair of homologous chromosomes spatially position themselves in relation to the centrosome axis. The center of volume of the centrosomes by γ-tubulin immunofluorescence staining defined the *x*-axis and was used for alignment of metaphase cells.

### Defining the relative position of homologous chromosomes

The centers of volume for the 3D reconstructed surfaces of homolog chromosome paint signals and DNA counterstains were obtained via the statistics tool in Imaris. 3D reconstructed homologs were color-coded as either colored (cyan, magenta, red, yellow, or green) or gray depending on their distance from the *x*-axis (*y*=0). The homolog that had the *y*-value closest to *y*=0 would be designated as the colored homolog and the other respective homolog would be gray. Furthermore, the cells were oriented so that colored homologs occupied the more positive positions than the gray homologs. The position of each homolog was analyzed using the ‘position y’ statistic, which provided the *y*-coordinate of the center of volume of the 3D surface.

The position of each pair of homologous chromosomes was normalized in every mitotic cell with regards to the DNA counterstain (similar to the process in Hua and Mikawa, 2018b). The relative positions of each homologous chromosome was calculated by normalizing the position of the y-coordinate of the homolog to the total length of the DNA metaphase plate along the *y*-axis (BoundingBoxAA Length Y) according to the following equation:

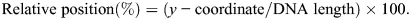
The normalization scales chromosome positions to a range of approximately −50% to +50%, corresponding to the lower and upper halves of the metaphase plate, respectively. For example, a chromosome 19 homolog in HUVECs had a *y*-coordinate of 2.71 μm and a DNA metaphase plate length of 9.12 μm. The relative position was therefore calculated as (2.71/9.12)×100=29.7%.

Immunofluorescence staining for γ-tubulin, along with a DNA counterstain, revealed that the centrosome axis and the volume-derived axis positioned perpendicular to the longest axis of the metaphase DNA plate was an average distance of 0.5 μm apart (Fig. S1). Consequently, individual chromosome positions within ±0.5 μm of the DNA center of volume, referred to as the ‘boundary region’, were excluded from the analysis. For instance, out of 66 total cells, dix cells exhibited homologous chromosome 1 in this boundary region, rendering the data uninterpretable (*n*=6/66 cells for chromosome 1).

### 3D distance and angular orientation measurements of homologous chromosomes

Homologous chromosomes were identified as paired if the distance between them was 0 μm (≤0.36 μm, the spatial *xy* resolution limit of the confocal microscope) and an angular orientation (vector angle) of 0°.

Minimal distances between homologs were determined by the shortest distances between the edges of the 3D surfaces of homologs.

The 3D vector coordinates determined the angular orientation of the homologs. To determine a 3D vector coordinate, a 3D surface of a homolog was selected. The longest ellipsoid axis length (ellipsoid axis C) was identified in the ‘specific values’ tab. The *x*, *y* and *z* coordinates were then acquired to define the 3D vector. This process was repeated for the other homolog. The vector angle was calculated by taking the inverse cosine of the normalized dot product.

At metaphase, it was difficult to determine the centromere position in relation to the chromosome arm due to observing a fixed image when centromeres oscillate along the centrosome axis and chromosome arms are more dynamic. Therefore, homolog orientation was quantified using a single quadrant (90° range) instead of the 180° range that was previously used for 3D vector angle analysis of anaphase cells in which the vectors had a defined direction (Hua and Mikawa, 2018b).

### Statistical analysis

The loss of spatial segregation was used to describe the position of individual homologs across the centrosome axis. The loss of antipairing was used to describe the loss of haploid set organization. Throughout the manuscript, we used these terms to describe the loss of statistically significant non-random spatial segregation, rather than the complete elimination of homolog separation. Our definition was based on statistical testing against the null hypothesis that homologous chromosomes were positioned randomly with respect to one another, similar to nonhomologous chromosome pairs (Hua and Mikawa, 2018).

Pairing was defined as occurring in cells where homologs had a distance and angular orientation difference of 0. Contingency table analysis or binomial logistic regression was used to assess the effect of explanatory factors on the probability of pairing. Statistical analysis was completed using the statistical analysis software, JMP Pro 18 (SAS Institute, Raleigh, NC).

### Data visualization

All figures were generated using Adobe Photoshop (Version 26.3.0). Bar graphs depicting pairing frequencies of chromosomes between HUVECs and HAECs were generated with Graphpad Prism 11 [Version 11.0.2 (92)].

Rotational video of both single whole chromosome painting and 3D overlays for HUVECs and HAECs were generated using the video editing software, Adobe Premiere Pro (Version 25.0.0, Build 61). To show side-by-side comparison of chromosome positioning of both HUVECs and HAECs, each cell was repositioned and resized using the position tool for both *x* and *y*, and the scale function within the properties tab. The text was implemented in the video using the type tool within the tools tab and the corresponding text colors were changed within the properties tab. Video was exported and used the preset ‘Match Source - Adaptive High Bitrate, Format of H.264 and recorded at 24 fps’.

## Supplementary Material



10.1242/joces.264668_sup1 Supplementary information
