# Supplementary material for: Higher frequency of homologous chromosome pairing in adult endothelial cells as compared to in neonatal endothelial cells
Source: J Cell Sci. 2026 Oct 23;139(20):jcs264668. doi: 10.1242/jcs.264668 (PMC13723388; doi:10.1242/jcs.264668)
Supplement: Supplementary file 2 [file joces-139-264668_review_history.pdf]

Higher frequency of homologous chromosome pairing in adult endothelial cells as compared to in neonatal endothelial cells

Jemery Morales, Omar Akhtar, Gabriel Quintero Plancarte and Lisa Hua
DOI: 10.1242/jcs.264668

Editor: Megan King

Review timeline
Original submission: 17 December 2025
Editorial decision: 12 March 2026
First revision received: 7 August 2026
Editorial decision: 16 September 2026
Second revision received: 22 September 2026
Accepted: 23 September 2026

Original submission

First decision letter

MS ID#: jcs.264668

MS TITLE: Higher frequency of homologous chromosome pairing in human adult aortic endothelial cells

AUTHORS: Jemery Morales; Gabriel Quintero Plancarte; Lisa L Hua

ARTICLE TYPE: Research Article

Dear Lisa,

Thank you for your considerable patience while we worked to secure expert reviews for your manuscript. We have now reached a decision.

As you will see, the reviewers gave favourable reports but raised some critical points that will require amendments to your manuscript. While these comments are extensive, they focus almost entirely on clarifying the analyses, methods, and text, with few experimental requests. I hope that you will be able to carry these out because I would like to be able to accept your paper, depending on further comments from reviewers.

Reviewer 1

SUMMARY OF THE ADVANCE MADE IN THIS PAPER AND ITS POTENTIAL SIGNIFICANCE TO THE FIELD

Anti-pairing, or spatial separation of homologous chromosome sets during metaphase was reported for neonatal human umbilical vein endothelial cells (HUVEC) but whether this chromosome configuration exists in other cell types was unknown. Here the Hua group has carried out a careful study of the extent this phenomenon in adult aortic endothelial cells (HAECs) and compared data to HUVEC cells analyzed in parallel. The manuscript is organized into various chromosome sets (e.g. 17 and 19; acrocentric chromosomes; large chromosomes 1 and 4 and sex chromosomes in XX and XY individuals. In all cases a the HAECs showed loss of segregation and elevated levels of abnormal homolog pairing. One strength of the study is the use of cells from different donors of different ages. These findings suggest that mechanisms governing chromosome antipairing may decline with

© 2026. Published by The Company of Biologists under the terms of the Creative Commons Attribution License (https://creativecommons.org/licenses/by/4.0/).

aortic endothelial cell age. The work is the first to link decreased antipairing previously seen in human umbilical vein endothelial cells to adult endothelial cells.

SUGGESTIONS TO AUTHORS

Major comments
Overall, the analysis is highly rigorous, the outcomes are clear, and the data support the conclusions. I think the discussion could be dramatically streamlined to cut out mention of future experiments and speculation since it is peripheral to the manuscript. My comments are primarily focused on changes that could improve the clarity of the presentation of data and some confusing wording.

1. Abstract: "During mitosis, pairing of homologous chromosomes can be detrimental and has been correlated with gene misregulation, chromosomal aberrations, and various pathological diseases." I am not sure this has been rigorously addressed for mitosis. This statement should have supporting evidence in the introduction.
2. The manuscript presents a comparison; however, the title employs comparative language without clearly identifying the compared conditions. Also the title does not indicate that pairing is a pathological state. Focusing on the loss of spatial separation of parental genome sets would be more descriptive.
3. Page 12, line 33 "HAECs are typically sourced.." I think it would be most transparent to describe the patient set before describing individual chromosome and make it clear that not all chromosome sets being analyzed for each patient. Line 3 of this paragraph indicates n=4 patients for chromosomes 17 and 19, but then five patients are noted, but actually there are six total according to the supplement.
4. It is stated in the discussion that the samples are from patients undergoing surgery for hypertension. I think this should be stated up front.
5. Page 12, line 48 " To determine whether small chromosomes". The paragraph states n=82 and n=38 but how many have lost the pattern? (The HUVEC example states n = 17/17), can the same format be used here?
6. The section headings and figure legend titles should be uniform. It is confusing to say that "Fewer adult HAECs show loss of segregation for the larger chromosomes 1 and 4" (page 14 line 6) without stating what is being compared. Same is true for the section on sex chromosomes: "Few female adult HAECs exhibit loss of X chromosome segregation and abnormal pairing as compared to HUVECs" (page 19 line 12) isn't there more loss of X chromosome segregation? Perhaps the wording throughout the manuscript can be harmonized so that the data is reported similarly.
7. I like the summary figure 5. It would be nice to see a summary of the frequency of segregation loss for each chromosome/patient. Also since not all chromosomes were analyzed for all patients, the chart should somehow indicate this (e.g. N/A as in Supplemental Table 1). I am wondering if it is misstated on page 28 line 20 since Fig. 5b reports on homolog pairing not loss of spatial segregation.
8. Page 28 "These observations suggest that the potential for sex chromosomes, especially the Y chromosome, to behave differently from autosomes during development and aging." There does not seem to be evidence X chromosomes behave differently than autosomes in terms of segregation loss so perhaps this sentence might be qualified as sex chromosomes in males.
9. Page 28 The last sentence of section on samples (top of page 28) "Additional data is. necessary…." can be deleted.
10. Page 28, "This suggests loss of antipairing is conserved.." To avoid genetic comparisons, I might just say it is a common feature.
11. Page 28 "graphical data suggests a preliminary age-dependent loss…" the data in 5B shows that the oldest individual has the highest degree of loss of spatial segregation. I would be hesitant to say there is an age-dependent loss since one of the 50 year old females had almost no loss. There does seem to be a trend in the increase in pairing frequency.
12. Page 28, I think you can delete the section on future studies and focus on the findings in the paper.
13. Page 29, line 13 I would delete this paragraph, the study covered several types of chromosomes, if there is a particular feature that was not included, then perhaps, but otherwise this paragraph is unnecessary.
14. Page 29 line 29, In Drosophila pairing is the norm, is it relevant to compare these systems?

© 2026. Published by The Company of Biologists under the terms of the Creative Commons Attribution License (https://creativecommons.org/licenses/by/4.0/).

15. Page 29 line 42 "Comparative studies…" I would delete this sentence since you already said in the sentence above that it is conserved in mouse and bovine, unless you want to speculate for other vertebrates, but I don't think this is necessary.
16. Page 30, line 9/10. I am not sure how relevant the discussion of spatial arrangement of chromosomes and gene expression is to mitotic antipairing since I was under the impression that homologs were more or less randomly distributed in nonmitotic cells and this organization is clearly lost in metaphase cells. I also do not see the connection between genomic instability and mitigating the consequences (of what?)

Minor comments
1. Figure 1. Text for panels A and S are very hard to read. Please consider a larger font size. The inset of the optical sections for part C are also very small
2. Figure 5b. Demarcating the regions of the chart showing data from each individual (e.g. with vertical dotted lines would be appreciated. It is very hard to tell from the ticks on the X axis and the position of the text where the boundaries are.
3. Page 26 line 4, remove extra comma

Reviewer 2

SUMMARY OF THE ADVANCE MADE IN THIS PAPER AND ITS POTENTIAL SIGNIFICANCE TO THE FIELD

This manuscript builds on the important findings reported in an earlier publication by the senior author, Lisa Hua (2018 PNAS PMC6310853). In that earlier publication, the authors reported that, in neonatal human umbilical vein endothelial cells (HUVECs), the two homologs of every chromosome pair largely reside on opposite sides of a hypothetical plane lying roughly along the centrosome axis at metaphase. This antipairing of homologs was proposed to protect the genome against abnormal mitotic recombination and genomic instability. In this paper, the authors investigate whether this previously observed phenomenon of mitotic antipairing persists in adult human endothelial cells (HAECs). They use ImmunoFISH combined with whole chromosome paints to visualize and map the positions of chromosomes 1, 4, 13, 15, 17, 19, 21, 22, X, and Y relative to the centrosome plane in HAECs from 6 donors ranging in age from 21 to 68. They then compared these positions to those representing HUVECs. They conclude the following:

A) Small chromosomes (13, 15, 17, 19, 21, 22) and XY showed reduced spatial segregation in adult HAECs, with 8-28%* of cells showing abnormal pairing compared to 0-3% in neonatal HUVECs. *Corrected from values given in the ms, see Comments, below.

B) Compared to the smaller chromosomes, large chromosomes (1, 4) and XX showed less spatial segregation and abnormal pairing (3-8%). The authors interpret this to mean a size-dependent, gradual decline in antipairing fidelity.

C) The X and Y chromosomes also showed reduced spatial segregation but did not exhibit pairing, which the authors attribute to minimal sequence homology between X and Y.

D) Abnormal pairing was observed across all 6 patients, suggesting a conserved age-related phenomenon rather than patient-specific variation.

E) The authors speculate that the differential loss of antipairing may relate to interchromosomal contacts among acrocentric chromosomes involved in nucleolus assembly as well as the tendency of smaller chromosomes to localize centrally along the centrosome axis, making them more susceptible to loss of spatial segregation.

This study constitutes a valuable contribution to our understanding of 3D genome organization and the potential role of antipairing in maintaining genome stability. The observations are thought-provoking and, as antipairing is a relatively new concept, this study could be especially impactful. Furthermore, the use of patient samples (as versus cell culture) makes the study directly meaningful in terms of human health. The manuscript, however, will need to be revised prior to publication, as there are many points in need of clarity and additional background/information as well as conclusions that would best be toned down. In addition, there are inconsistencies between the text and figures as well as within and between figures in terms of data. While we do not

© 2026. Published by The Company of Biologists under the terms of the Creative Commons Attribution License (https://creativecommons.org/licenses/by/4.0/).

believe the correction of these inconsistencies will change the take-home messages, it will be important for the authors to revisit their conclusions after the corrections are in place.

SUGGESTIONS TO AUTHORS

1) We encourage the authors to include more information about the probes, even if commercially obtained. For example, are the probes expected to cover the entire length of the chromosomes? Do they target repeated sequences, such as centromeres, telomeres, rDNA, and/or interspersed repetitive elements?

2) We assume that the authors did not synchronize their cell cultures but, given the focus on metaphase chromosomes, it would be helpful if the authors made an explicit statement on this point.

3) We encourage the authors to explain angular orientation in greater detail both in the text as well as in Figure 1. For example, was this determined in 3D space, or did the authors project the homologs onto a metaphase plane? Furthermore, should readers assume that the authors disregarded the p and q arm orientation of the homologs when determining angular orientation?

4) While we assume that the center of mass for individual chromosomes and for the "chromosomal mass" (which we assume refers to the entire genome) is based on density rather than volume, it will be important to clarify this in the Results and Material and Methods.

5) We recommend that the information about the "boundary zone" as presented in Supplemental Figure 1 be included in the main text. Not only will readers have a better understanding of how the axes were drawn, the authors will have an opportunity to resolve concerns as to whether the discounting of chromosomes (e.g. 6/66 cells in the case of chromosome 1) could have affected outcomes.

6) So that readers can better understand the breadth of sizes and configurations for homolog pairs, we suggest the authors select one chromosome and then show all the HUVEC and HAEC segmented images one cell at a time (e.g., in the spirit of Supplemental Figure 2 a'''). Such a montage would be quite compelling in terms of the authors' goals.

7) The authors say they chose to study chromosomes 17 and 19 because of their differences in basepair number, but other pairs of chromosomes (even if selected just from the pool of smaller chromosomes) show a much greater difference in size. While becomes clear later in the narrative that the authors wanted to avoid chromosomes carrying rDNA for the first part of their narrative, it remains unclear why they did not consider, for example, chromosomes 16, 18, or 20. Perhaps the authors could provide more detail for their reasoning.

8) We are curious why the authors did not study chromosome 14, as it also carries rDNA. (We do not at all mean to suggest that chromosome 14 needs to be included in the story.)

9) We appreciate the authors providing average distances and average angular orientations, but including this information in the legends makes it difficult to compare these values across all experiments. Might the authors consider putting all of this information in one or two tables?

10) The Materials and Methods indicate that HUVECs were commercially obtained in two samples, each representing 10 individuals, while the Results points out that experiments using HUVECs used a pool of 20 individuals. We suggest the authors clarify in the Results whether the two samples were pooled prior to culturing and, if not, which data were obtained from which sample. It would also be important to mention in Results that HUVECs were commercially obtained.

11) The data for HAECs are derived from 6 donors, but it is not clear in Figures 1 - 4 which data came from which donor. While some information could be extracted from Figure 5, it is time-consuming to do so, with outcomes being potentially ambiguous. Furthermore, due to the extensive use of color coding, the information in Figure 5 will not be fully accessible to readers who are color-blind. In short, we recommend the authors include this information in additional table(s). We

© 2026. Published by The Company of Biologists under the terms of the Creative Commons Attribution License (https://creativecommons.org/licenses/by/4.0/).

also recommend that Figure 5b be annotated along the x axis so that chromosome and patient identity are unambiguous, with tick marks placed even when the pairing frequency is zero.

12) Would it be possible to have the authors comment on why the number of samples per chromosome is quite different between HUVECs and HAECs? For instance, for chromosome 17, the cell counts are 12 versus 82 for HUVECs and HAECs, respectively.

13) We assumed that the differences in Figure 5b between HAEC samples are due largely to a stochasticity inherent in the system but, in light of all adult donors being associated with hypertension (was this true of all adult donors?), we are curious about other health-related characteristics of the donors. Thus, we suggest that all potential relevant health-related information be included in Results at the time that HAECs are first mentioned (or, if there is no additional information to make this clear). Here, too, we recommend that the commercial source for HAECs be mentioned in the Results.

14) The authors mention the potential of aortic cells to be aneuploid. Did they encounter aneuploidy in their samples and, if so, how were such instances handled in terms of data acquisition and quantification?

15) The Introduction does not provide sufficient background for haploid sets and antipairing. This is especially important because most readers will not be well-informed about these topics. Where else have haploid sets and antipairing been observed and, as importantly, where they have not been observed? The more readers know prior to reading the Results, the more easily they will recognize the advance of the authors over prior work. Thus, we recommend the authors move background information about haploid sets and antipairing from the Discussion ("Segregation of haploid genomes, or antipairing, has also been observed...to maintain overall genomic stability.") to the Introduction as well as provide considerably more detail. That haploid set organization defines the earliest stages of development (Reichmann et al. 2018, Mayer et al. 2000a,b,) makes it even more impactful that the authors observe a degradation of this pattern postnatally. The earlier readers are aware of this, the more exciting the Results will be. Similarly, knowing early on in the manuscript that antipairing may be an important process in Drosophila (Joyce, et al. 2012) will emphasize how the authors' findings likely extend across species - this is exciting! In brief, we urge the authors make better use of the Introduction to set the stage for their story. If word limits are an issue, we would recommend the authors shorten the more general introduction to 3D genome organization.

Additional references to be included are:
Nagele et al. 1995 (PMID: 8525379) with respect to haploid sets
Nagele et al. 1998 (PMID: 9880766) with respect to haploid sets
Joyce et al. 2016 (PMC4939289) with respect to antipairing
Stamatov et al. 2025 (PMC11991918), a recent example where haploid sets and antipairing are not observed.

16) We believe the authors may have intended to cite Keneklis and Odartchenko 1973 (PMID: 4693953) rather than the 1974 paper by the same authors, as the latter is primarily a "control" for the former. Citing both references would also be fine.

17) "Loss of spatial segregation" and "loss of antipairing" could mean a complete absence of these processes, which we believe is a more extreme interpretation than intended. Perhaps the authors would consider alternative phrasing.

18) The proposal of a potential age-related impact on antipairing is interesting but, owing to the small sample size of adult donors (6) and undersampling of neonatal material in terms of fetal age and tissue type, we encourage the authors to be more conservative in their interpretations. Phrases that are problematic include (but are not limited to): "may decline with aortic endothelial cell age, leading to increased susceptibility to abnormal pairing and cardiovascular disease (Abstract, page 3,)", "trend of increased susceptibility of pairing throughout age (subtitle and figure legend, pages 22 and 25, respectively)", and "after birth, there may be an increased susceptibility (narrative, page 22)". A more conservative tone can be achieved through rewording statements, reminding readers of the small sample sizes and thus tentativeness of the proposals, and moving statements

© 2026. Published by The Company of Biologists under the terms of the Creative Commons Attribution License (https://creativecommons.org/licenses/by/4.0/).

to the Discussion. These changes would be in line with the authors' acknowledgment of limitations in the Discussion and proposal of the kinds of concrete future experiments that are needed.

19) Similarly, the authors discuss a potential correlation between the reduced frequency of antipairing and cardiovascular disease but do not provide functional evidence connecting the two. This angle would benefit from more cautious framing.

20) As the definition of "pairing" is fairly stringent and rigid, we wonder whether such strictness could have led the authors to miss subtle patterns. In other words, would a window of distance/angle thresholds (rather than a simple cut-off) capture a gradient of pairing disruption and, from there, additional discoveries? Although we are curious whether this approach will allow the authors to glean additional insights, we are not requiring that this approach be taken.

21) Do the authors have direct (or even indirect) evidence confirming that their samples represent normal karyotypes? Along these lines, did the authors come across situations where only one homolog was visible? If so, how did the authors distinguish monosomy from an instance of pairing that was extensive enough to preclude the distinction of two homologs?

22) We are curious whether the authors took other features of the homologs into account (e.g., chromosome shape and size) and, if so, were there any correlations between these features and antipairing?

23) We discovered several discrepancies within figures, between figures, and between the text and figures (see below) and, as we only spot checked the manuscript, there may be other discrepancies in the manuscript. We urge the authors to doublecheck all their data and calculations, including their statistical tests. Examples of what we noticed:

a) Figure 1n reports 83 cells, while Figure 5a reports only 82.
b) Figure 1s indicates a total of 99 cells for chromosome 17, while Figure 5a notes 94. As would be expected, the two P values for these figures also differ.
c) Figure 2y indicates a 0.29 pairing frequency for chromosome 15 in HAECs, while Figure 2r would indicate a frequency of 0.28 (14/50).
d) On page 18 (lines 44-50), the authors compare the pairing frequency of the larger chromosomes (1, 4) with that of the acrocentric chromosomes (13, 15, 21, 22), citing a total of 320 cells. However, Figure 2y reports a total of only 200 HAEC cells for acrocentric chromosomes. We believe the total of 320 includes the data for non-acrocentric small chromosomes 17 and 19. As such, the frequency of pairing, will change, as will the outcome of the chi-square test.
e) On page 18 (lines 50-55), the authors report a pairing frequency of 14.18% for chromosomes 13, 15, 17, 19, 21, and 22 (61/430), but we believe 430 should be replaced with 320, as 430 likely includes the cell counts for chromosomes 1 and 4 as well. Furthermore, we feel the count of 61 overstates the number of paired homologs. (Note, if the total HAECs with respect to chromosome 17 is actually 83 instead of 82, then the correct total should be increased by 1 to 321.)
f) The counts for XY HAEC cells in Figures 4l and 4m are 44, while Figure 5a reports 43.

Minor comments
We have a number of suggestions for enhancing the clarity of the figures and tables:
1) We encourage the authors to increase the font size for Figures 1a and 1s and for Figure 2, throughout. Please also increase the size of the red P in Figures 1- 4.

2) Figure 1b: Would it be possible to reorder the nuclei to match the order of the data in the graph below?

3) Figures 1d and 1e: The color scheme and how homologs were designated one color or the other is not immediately clear. One solution would be to move Supplementary Figure 2 into the main text.

4) We suggest Fig. 1s conform to the style of Fig. 2y, 3m, and 4m, enumerating sample size for each assay.

© 2026. Published by The Company of Biologists under the terms of the Creative Commons Attribution License (https://creativecommons.org/licenses/by/4.0/).

5) We recommend that the number of paired homologs be given above the bars in Figures 1s, 2y, 3m, 4m, 5a, and 5b, as the y-axis is already providing pairing frequencies. We suggest this because we often found ourselves back calculating the number of paired homologs.

6) The data points are too difficult to distinguish in Figures 1n, 2x, 4i. One solution would be to put some of the cell numbers outside the mass of datapoints, connecting them to their nucleus with lines.

7) Figure 5b: We suggest the authors remove "Groups" from title because this term could imply that there were multiple donors for the younger and older ages.

8) Supplemental Table 1: This table can be simplified by removing "n=" and "cells" wherever possible.

First revision

Author response to reviewers' comments

Please find our revised manuscript attached (#jcs.264668). We would like to thank both reviewers for their helpful comments and constructive criticisms to improve our manuscript. We have incorporated all of their suggestions. We believe these changes have significantly strengthened the manuscript. Our point-by-point responses to each of the reviewer's comments are provided below.

Reviewer #1

We thank Reviewer 1 for the careful evaluation of our manuscript and for the thoughtful, constructive comments and suggestions. We have carefully considered each point and have revised the manuscript in response. These revisions include the addition of new data (Fig. 4, Supplemental Fig. 3, and Supplemental Tables 2-4), clarification of the analyses, and revisions throughout the text to improve accuracy, and transparency.

Major Points:

Critique 1. *Abstract: "During mitosis, pairing of homologous chromosomes can be detrimental and has been correlated with gene misregulation, chromosomal aberrations, and various pathological diseases." I am not sure this has been rigorously addressed for mitosis. This statement should have supporting evidence in the introduction.*

Response:
We thank the reviewer for this thoughtful comment. We agree that the statement was too broad, as the detrimental consequences of homologous chromosome pairing have not been rigorously established specifically during mitosis. To address this concern, we have removed the phrase "During mitosis" from the abstract to avoid overstating the current evidence. The revised statement is now more accurately aligned with the existing literature.

Critique 2. *The manuscript presents a comparison; however, the title employs comparative language without clearly identifying the compared conditions. Also the title does not indicate that pairing is a pathological state. Focusing on the loss of spatial separation of parental genome sets would be more descriptive.*

Response:
We thank the reviewer for this suggestion. In response, we have revised the title to clearly identify the two cell types being compared: **"Higher Frequency of Homologous Chromosome Pairing in Human Adult Aortic Endothelial Cells Compared to Neonatal Umbilical Vein Endothelial Cells."**

We chose not to characterize homologous chromosome pairing as a pathological state in the title

© 2026. Published by The Company of Biologists under the terms of the Creative Commons Attribution License (https://creativecommons.org/licenses/by/4.0/).

because, although there are studies reporting correlations between increased homolog pairing and human disease associated phenotypes, the available evidence does not establish a direct causal relationship. Therefore, we did not wish to overstate our findings. We also appreciate the suggestion to focus on the loss of spatial separation of parental genome sets. While this is an intriguing model for haploid genome organization, our current study does not provide direct evidence to support this mechanism. Accordingly, we selected a title that accurately reflects the data presented without extending beyond the conclusions supported by our results.

Critique 3. *Page 12, line 33 "HAECs are typically sourced.." I think it would be most transparent to describe the patient set before describing individual chromosome and make it clear that not all chromosome sets being analyzed for each patient. Line 3 of this paragraph indicates n=4 patients for chromosomes 17 and 19, but then five patients are noted, but actually there are six total according to the supplement.*

Response:
We thank the reviewer for identifying this point of confusion. To improve clarity and transparency, we have revised the Results section to first describe the donor cohort before presenting the chromosome-specific analyses. The revised text now states: "*HAECs were sourced from individual deceased organ donor patients of various ages, and purchased commercially (Lonza). Some donors had pathologies such as hypertension (n=2/7 patients). For example, one of the 50-year-old females reported hypertension and glioblastoma, while the other 50-year old-female reported fibromyalgia and acute alcohol-related pancreatitis. The 68-year-old male reported hypertension, hyperlipidemia, and diabetes. The other four remaining patients did not report pathologies. In addition, not all patients were analyzed for each chromosome.*"
We also appreciate the reviewer noting the discrepancy in the number of donors analyzed. To address this, we included an additional 45-year-old female donor in the study and revised both the manuscript text and Supplemental Table 1 to explicitly indicate which donors were analyzed for each chromosome. The revised Results section now reads: "*To investigate whether this pattern is present across multiple patients, we analyzed HAECs at metaphase from separate patients (Supplemental Table 1). For chromosome 17 analysis, HAECs from the 36-year-old female, both 50-year-old females, and 68-year-old male were analyzed (Supplemental Table 1, n=4 patients). For chromosome 19, HAECs from the 36-year-old female, 50-year-old male, one of the 50-year-old females, and 68-year-old male were analyzed (Supplemental Table 1, n=4 patients). Between chromosomes 17 and 19, HAECs were analyzed from 5 patients total (Supplemental Table 1).*"

Critique 4. *It is stated in the discussion that the samples are from patients undergoing surgery for hypertension. I think this should be stated up front.*

Response:
We thank the reviewer for this comment. We revisited the source of the HAEC samples and contacted the commercial vendor (Lonza) to verify the donor information. Based on the information provided by Lonza, it was not possible to confirm that the samples were obtained from patients undergoing surgery for hypertension. Rather, the HAECs were isolated from deceased organ donors.

To ensure the manuscript accurately reflects the sample source, we have removed the statement from the Discussion indicating that the samples were from patients undergoing surgery for hypertension. We have also revised the Results section to clearly describe the origin of the HAECs, including that they were commercially obtained from Lonza and derived from deceased organ donors, along with the available donor demographic and clinical information. These revisions provide a more accurate and transparent description of the study samples early in the manuscript.

Critique 5. *Page 12, line 48 " To determine whether small chromosomes". The paragraph states n=82 and n=38 but how many have lost the pattern? (The HUVEC example states n = 17/17), can the same format be used here?*

Response:
We thank the reviewer for this helpful suggestion. To improve clarity and make the presentation of the data consistent with the HUVEC example, we have revised the text to explicitly report the number of cells that retained the pattern of one homolog per nuclear hemisphere. In addition, Supplemental Table 1 summarizes the underlying cell counts for each chromosome analyzed. The

© 2026. Published by The Company of Biologists under the terms of the Creative Commons Attribution License (https://creativecommons.org/licenses/by/4.0/).

revised text now reads: "*To determine whether small chromosomes maintain the spatial segregation of homologs in adult HAECs, we analyzed chromosomes 17 and 19 using ImmunoFISH/WCP. We found that both chromosomes 17 and 19 lose the pattern of one homolog per nuclear hemisphere in HAECs derived from five patients (Fig. 1 k-r segregation in=44/83 cells for chromosome 17; segregation in n=21/37 cells for chromosome 19, Supplemental Fig. 3B, Supplemental Tables 1 and 2).*"

Critique 6. *The section headings and figure legend titles should be uniform. It is confusing to say that "Fewer adult HAECs show loss of segregation for the larger chromosomes 1 and 4" (page 14 line 6) without stating what is being compared. Same is true for the section on sex chromosomes: "Few female adult HAECs exhibit loss of X chromosome segregation and abnormal pairing as compared to HUVECs" (page 19 line 12) isn't there more loss of X chromosome segregation? Perhaps the wording throughout the manuscript can be harmonized so that the data is reported similarly.*

Response:
We agree that the section headings and figure legend titles should be presented in a consistent manner and clearly identify the comparison being made. In response, we have revised the section headings and figure legend titles throughout the manuscript to improve consistency and clarity.

Specifically, we revised the section heading to read:" *Fewer adult HAECs show loss of segregation for the larger chromosomes 1 and 4 as compared to neonatal HUVECs*", which explicitly identifies the two cell types being compared.

We also revised the section on sex chromosomes to avoid ambiguity. The heading now focuses on the principal observation of our study: "*Female adult HAECs showed an increase in abnormal pairing of XX chromosomes as compared to XY chromosomes in male HAECs*." This wording more accurately reflects the data presented and is consistent with the terminology used throughout the manuscript.

Critique 7. *I like the summary figure 5. It would be nice to see a summary of the frequency of segregation loss for each chromosome/patient. Also since not all chromosomes were analyzed for all patients, the chart should somehow indicate this (e.g. N/A as in Supplemental Table 1). I am wondering if it is misstated on page 28 line 20 since Fig. 5b reports on homolog pairing not loss of spatial segregation.*

Response:
We thank the reviewer for this thoughtful suggestion and for the positive comments regarding the summary Figure 5. To improve the presentation of the data, we have added Supplemental Table 2, which summarizes the frequency of loss of spatial segregation for each chromosome and each patient analyzed. This table provides a comprehensive overview of the results across all donors.

In addition, to clearly indicate chromosomes that were not analyzed for specific patients, we have revised Supplemental Table 1 to include "N/A" for chromosomes not tested.

We also appreciate the reviewer identifying the inconsistency in the manuscript text. The statement on "page 28, line 20" has been corrected to accurately reflect the data presented in Figure 5B, which summarizes homologous chromosome pairing rather than loss of spatial segregation.

Critique 8. *Page 28 "These observations suggest that the potential for sex chromosomes, especially the Y chromosome, to behave differently from autosomes during development and aging." There does not seem to be evidence X chromosomes behave differently than autosomes in terms of segregation loss so perhaps this sentence might be qualified as sex chromosomes in males.*

Response:
We agree that our original statement was unclear regarding sex chromosomes as a group. In response, we have removed the sentence from the manuscript. As we further considered our findings, we also noted that chromosome 21 exhibits behavior distinct from several other autosomes, indicating that the observed differences are not unique to the sex chromosomes. To more accurately reflect the available evidence, we revised the Discussion to focus specifically on the Y chromosome, where there is literature supporting its unique susceptibility to genomic instability during aging.
The revised text now reads: "*Consistent with this, the Y chromosome also undergoes frequent*

© 2026. Published by The Company of Biologists under the terms of the Creative Commons Attribution License (https://creativecommons.org/licenses/by/4.0/).

loss in aging tissues, particularly in the hematopoietic and blood cell lineages (Aviv et al. 2001; Gutiérrez-Hurtado et al. 2024; Jacobs et al. 1963; Miyai et al. 2008; Nath et al. 1995; Pierre and Hoagland 1972; Sakurai and Sandberg 1976; Stone and Sandberg 1995; Weng et al. 2016; Wiktor et al. 2000; Zhang et al. 2007). It may be uniquely vulnerable to genomic instability due to its structure, limited recombination, and small size (Colaco and Modi 2018; Rogers 2021)."

Critique 9. *Page 28 The last sentence of section on samples (top of page 28) "Additional data is. necessary…." can be deleted.*

Response:
We agree that this statement was unnecessary and have removed the final sentence from the section as recommended.

Critique 10. *Page 28, "This suggests loss of antipairing is conserved.." To avoid genetic comparisons, I might just say it is a common feature.*

Response:
We thank the reviewer for this helpful suggestion. We agree that the term "conserved" could imply a genetic comparison that is not supported by our data. Accordingly, we have revised the sentence to avoid this confusion. The text now reads: "*This suggests the loss of antipairing is a common feature in adult aortic ECs (Fig. 5 c).*"

Critique 11. *Page 28 "graphical data suggests a preliminary age-dependent loss…" the data in 5B shows that the oldest individual has the highest degree of loss of spatial segregation. I would be hesitant to say there is an age-dependent loss since one of the 50 year old females had almost no loss. There does seem to be a trend in the increase in pairing frequency.*

Response:
We thank the reviewer for this comment. We agree that our original statement may have overstated the relationship between age and the loss of spatial segregation. As the reviewer notes, there is variability among donors, including one 50-year-old female who exhibited relatively little loss of spatial segregation. We have revised the text to avoid implying an age-dependent effect that is not supported by our current dataset.

The revised sentence now reads: "*Nonetheless, graphical data suggests a preliminary age-based loss of spatial segregation of homologous chromosomes, with a trend in the increase of pairing frequency during adulthood (Fig. 5 b).*"

Critique 12. *Page 28, I think you can delete the section on future studies and focus on the findings in the paper.*

Response:
We agree that the Discussion is strengthened by focusing on the findings presented in this study. We have removed the section describing future studies and revised the Discussion to emphasize the results and their interpretation.

Critique 13. *Page 29, line 13 I would delete this paragraph, the study covered several types of chromosomes, if there is a particular feature that was not included, then perhaps, but otherwise this paragraph is unnecessary.*

Response:
We thank the reviewer for this suggestion and have removed the paragraph from page 29 discussing the subset of chromosomes analyzed.

Critique 14. *Page 29 line 29, In Drosophila pairing is the norm, is it relevant to compare these systems?*

Response:
We agree that somatic homolog pairing is the normal state in *Drosophila* and that it is important not to imply a direct equivalence between the *Drosophila* and mammalian systems. However, we believe that discussing *Drosophila* is relevant because studies in this model have identified active

© 2026. Published by The Company of Biologists under the terms of the Creative Commons Attribution License (https://creativecommons.org/licenses/by/4.0/).

regulators that both promote and antagonize homolog pairing, providing important mechanistic insights into the regulation of homolog interactions. These findings suggest that antipairing is an actively regulated biological process rather than simply the absence of pairing. In addition, Reviewer #2 specifically requested that we expand the Introduction to provide greater background on haploid genome organization and antipairing, including examples from other model organisms. To address this request, we moved much of the discussion on antipairing from the Discussion to the Introduction and expanded the background to better define the current understanding of antipairing and its biological significance. We have revised the Introduction to better explain the rationale for including these studies and to avoid implying a direct equivalence between the *Drosophila* and mammalian systems.

The revised Introduction now reads: "*Antipairing may be an important process in the fruit fly, as it may be inhibited or enhanced by different regulators, suggesting there are mechanisms driving the antipairing of homologous chromosomes in different animal models (Joyce et al. 2012; Joyce et al. 2016). For example, in the early fruit fly embryo, a high-throughput FISH-based screen identifies genes involved in promoting or antagonizing somatic homolog pairing (Joyce et al. 2012). This finding suggests that there may be active antipairing mechanisms preventing the pairing of chromosomes in mammals. Taken together, these findings support the concept that antipairing is a biologically significant nuclear architecture strategy to maintain overall genomic stability*."

Critique 15. *Page 29 line 42 "Comparative studies…" I would delete this sentence since you already said in the sentence above that it is conserved in mouse and bovine, unless you want to speculate for other vertebrates, but I don't think this is necessary.*

Response:
We agree that the final sentence in this section was redundant and have removed it from the Discussion. In addition, to improve the overall flow of the manuscript and in response to Reviewer #2's request for additional background on haploid genome organization and antipairing, we moved the discussion of comparative animal models from the Discussion to the Introduction. This provides readers with the necessary context before the Results and eliminates redundancy later in the manuscript.

The revised Introduction now reads: "*Segregation of haploid genomes has also been observed in other model systems including the mouse, bovine, rabbit, fruit fly, and nematode (Aguilera et al. 2020; Hua et al. 2022; Keneklis and Odartchenko 1974; Maddox et al. 2005; Mayer et al. 2000a, b; Reichmann et al. 2018; Schneider et al. 2021; Joyce et al. 2012; Joyce et al. 2016; Gondos et al. 1970). This haploid set organization defines the earliest stages of development, with the observation of parental genome separation in mammalian models (Schneider et al. 2021; Reichmann et al. 2018, Mayer et al. 2000a, b). For example, in mouse and bovine cells, dual mitotic spindles at fertilization separate the two parental sets (Reichmann et al. 2018; Schneider et al. 2021). In addition, 5-bromodeoxyuridine (BrdU)-labeled sperm show paternal genome separation in mouse zygotes which persist until the four-cell stage (Mayer et al. 2000b). Electron microscopy using uranyl acetate and lead citrate staining of fertilized rabbit ova show intact parental pronuclei adjacent to each other up to 21 hours after mating (Gondos et al. 1970). After fertilization in the fruit fly, gonomeric cell divisions show the maternal and paternal genomes in apposition and structurally separate (Callaini and Riparbelli 1996; Loppin et al. 2015). These findings suggest a conserved mechanism. An evolutionary persistence may reflect selective pressure to limit harmful recombination and promote nuclear compartmentalization. In this study, we observe a degradation of the haploid set pattern postnatally.*"

Critique 16. *Page 30, line 9/10. I am not sure how relevant the discussion of spatial arrangement of chromosomes and gene expression is to mitotic antipairing since I was under the impression that homologs were more or less randomly distributed in nonmitotic cells and this organization is clearly lost in metaphase cells. I also do not see the connection between genomic instability and mitigating the consequences (of what?)*

Response:
We agree that our original discussion did not clearly distinguish between mitotic antipairing and interphase chromosome organization, and that the connection to gene expression and genomic instability required additional clarification. Our intent was to highlight that one proposed function of mitotic antipairing is to minimize inappropriate homologous chromosome interactions during

© 2026. Published by The Company of Biologists under the terms of the Creative Commons Attribution License (https://creativecommons.org/licenses/by/4.0/).

cell division. Although the mechanisms linking mitotic antipairing to gene regulation remain unclear, studies have shown that abnormal homologous chromosome pairing in interphase can be associated with altered gene expression. For example, Koeman et al. (2008) reported that abnormal pairing of the short arm of chromosome 19 in renal oncocytoma cells correlated with misexpression of chromosome 19p genes involved in the hypoxia response. We recognize, however, that these observations are from interphase cells and should not be interpreted as direct evidence for a role of mitotic antipairing.

To address the reviewer's concern, we revised the Discussion to clarify that the evidence linking chromosome organization to gene expression is from interphase studies. The revised text now reads: “*Changes in chromatin structure further alter gene expression and increase susceptibility to DNA damage, with aging associated with higher levels of damage due to oxidative stress (Burgess et al. 2012; Wood et al. 2010; Wood and Helfand 2013; Zhang et al. 2021). Notably, the spatial arrangement of chromosomes within the cell may become disorganized in aged ECs, which can impact gene expression and overall cellular function (Ghosh and Meyer 2021; Heard and Bickmore 2007; Hua et al. 2022; Koeman et al. 2008; Lima et al. 2022; Misteli 2007). Aging disrupts nuclear organization through the loss of heterochromatin and nuclear lamina integrity, causing gene-rich regions to shift away from transcriptionally active domains, and altering gene expression patterns (Chandra and Kirschner 2016; Emerson and Lee 2023; López-Otín et al. 2013).*”

Minor points:

1. Figure 1. Text for panels A and S are very hard to read. Please consider a larger font size. The inset of the optical sections for part C are also very small

Response:
To improve the readability of Fig. 1, we have increased the font size in panels A and S. We have also enlarged the inset optical section in C to improve the visibility of the chromosome signals.

2. *Figure 5b. Demarcating the regions of the chart showing data from each individual (e.g. with vertical dotted lines would be appreciated. It is very hard to tell from the ticks on the X axis and the position of the text where the boundaries are.*

Response:
To improve the readability of Fig. 5B, we have added vertical dotted lines to clearly demarcate the data corresponding to each individual donor, as recommended.

3. *Page 26 line 4, remove extra comma*

Response:
The extra comma has been removed.

Reviewer #2
We thank and appreciate Reviewer 2 for the careful and insightful evaluation of our manuscript, as well as for the many thoughtful suggestions and encouraging comments. We greatly appreciate the reviewer's recognition of the significance of our work and the constructive recommendations that have strengthened the manuscript. In response, we have substantially revised the paper by expanding the Introduction, adding new data and analyses (including Fig. 4, Supplemental Fig. 3, and Supplemental Tables 2–4), clarifying the methodology, improving the presentation of the figures and tables, and revising the text throughout to improve clarity, accuracy, and interpretation. Our response to each specific comment is listed below.

Major Points:
Critique 1. *We encourage the authors to include more information about the probes, even if commercially obtained. For example, are the probes expected to cover the entire length of the chromosomes? Do they target repeated sequences, such as centromeres, telomeres, rDNA, and/or interspersed repetitive elements?*

Response:
We thank the reviewer for this helpful suggestion. To provide additional clarity regarding the

© 2026. Published by The Company of Biologists under the terms of the Creative Commons Attribution License (https://creativecommons.org/licenses/by/4.0/).

chromosome paint probes, we have expanded the Materials and Methods section to include details on their generation, genomic coverage, and hybridization characteristics.

The revised text now reads: "*Chromosome paint probes (ASI) are generated using flow-sorted chromosomes. DOP-PCR is used for DNA amplification with a semi-degenerate primer that anneals at random positions across the genome, with a bias towards primer binding site regions with more compatible 6-mer sequences. Thousands of DNA fragments are generated for each chromosome probe, ranging in size from approximately 200 bp to ~2,000 bp, with a peak distribution around 400–800 bp. The coverage of these fragments is broad but not uniform, with an estimation of ~70–85% coverage of the whole chromosome and similar for all chromosomes. Repetitive sequences such as centromeres and telomeres are underrepresented as they are blocked by Cot-1 DNA. The rDNA and X/Y Homologous Sequences can also display cross-hybridization artifacts and non-specific signals on the acrocentric and sex chromosomes.*"

Critique 2. *We assume that the authors did not synchronize their cell cultures but, given the focus on metaphase chromosomes, it would be helpful if the authors made an explicit statement on this point.*

Response:
We have clarified this point in the Materials and Methods section by explicitly stating that the cell cultures were not synchronized prior to analysis. We chose not to synchronize the cells to avoid introducing potential confounding effects associated with synchronization treatments.

We have added new text in Materials and Methods: *"HUVECs and HAECs were not synchronized prior to metaphase analysis to minimize potential confounding effects associated with cell synchronization procedures (Rodman et al. 1980; Rohlf et al. 1980)."*

Critique 3. *We encourage the authors to explain angular orientation in greater detail both in the text as well as in Figure 1. For example, was this determined in 3D space, or did the authors project the homologs onto a metaphase plane? Furthermore, should readers assume that the authors disregarded the p and q arm orientation of the homologs when determining angular orientation?*

Response:
We thank the reviewer for this thoughtful comment. To clarify how angular orientation was determined, we have substantially expanded both the Results and the Materials and Methods section. We now explain that homolog orientation was measured in 3D, rather than from a 2D projection, and clarify how vector angles were calculated.

We have also clarified that the orientation of the p and q chromosome arms were not used to determine angular orientation. Chromosome paint probes hybridize broadly across each chromosome and do not distinguish between p and q arm sequences.

The revised manuscript now includes the following text: "*Pairing is defined as having a 0 µm distance and a 0º vector angle, determined in a 3D space, between a pair of homologous chromosomes (Fig. 1 b). Thus, the spatial proximity of 0 µm distance and 0º vector angle is interpreted as direct spatial juxtaposition or contact along the axis of each homolog in our analysis (Fig. 1 b). P and q arm orientation was not used to determine angular orientation of homologs due to the resolution of the chromosome paint probes that bind to sequences across the chromosome without distinction of p and q regions.*

Also, at metaphase the centromere position is difficult to assign reliably in fixed cells because kinetochores undergo dynamic oscillatory movements while chromosome arms exhibit considerable flexibility (Cai, Casas, Quintero Plancarte et al. 2025; Walczak et al. 2010; Magidson et al. 2011). Consequently, vector directionality cannot be assigned with sufficient confidence. For this reason, we quantified homolog orientation using a 90° angular range, rather than the 180° range employed in our previous analyses of anaphase cells, where chromosome orientation is more clearly defined.
See new text: "*Our previous analysis of angular orientation was completed using a 180° range (Hua and Mikawa 2018b; Cai, Casas, Quintero Plancarte et al. 2025). Angular orientation is determined by the angle between the 3D vectors along each homolog. Each vector starts at the point of origin of the centromere, and runs through the chromosome arm via the longest ellipsoid axis. The vector angle was calculated by taking the inverse cosine of the dot product divided by the magnitude of the two 3D vectors. At anaphase, spindle microtubules attach to kinetochores that surround centromeres of individual homologs that pull sister chromatids apart*

© 2026. Published by The Company of Biologists under the terms of the Creative Commons Attribution License (https://creativecommons.org/licenses/by/4.0/).

(Walczak et al. 2010; Magidson et al. 2011). This leads to chromosome arms trailing behind the centromeres (Walczak et al. 2010; Magidson et al. 2011). At metaphase, the centromere position was less certain relative to the rest of the chromosome arm as centromeres/kinetochores oscillate parallel to the centrosome axis (Cai, Casas, Quintero Plancarte et al. 2025; Magidson et al. 2011). There is less constraint of chromosome arms at metaphase and more dynamic movement (Cai, Casas, Quintero Plancarte et al. 2025; Magidson et al. 2011). This stage difference makes it harder to identify the centromere position in fixed samples at metaphase, and does not allow us to assign directionality of vectors at the 180° range. Therefore, we limited our analysis to a single quadrant (90° range)."

We also expanded the Materials and Methods section to describe the 3D vector calculations, including how the longest ellipsoid axis of each chromosome was measured in Imaris and how vector angles were calculated using the inverse cosine of the normalized dot product. These additions provide a more complete description of the analytical approach and clarify the rationale for the modified angular analysis at metaphase.

See new text: "*The 3D vector coordinates determined the angular orientation of the homologs. To determine a 3D vector coordinate, a 3D surface of a homolog was selected. The longest ellipsoid axis length (Ellipsoid Axis C) was identified in the "specific values" tab. The x, y, and z coordinates were then acquired to define the 3D vector. This process was repeated for the other homolog. The vector angle was calculated by taking the inverse cosine of the normalized dot product.*

At metaphase, it was difficult to determine the centromere position in relation to the chromosome arm due to observing a fixed image when centromeres oscillate along the centrosome axis and chromosome arms are more dynamic. Therefore, homolog orientation was quantified using a single quadrant (90° range) instead of a 180° range that was previously used for 3D vector angle analysis of cells at anaphase in which the vectors had a defined direction (Hua and Mikawa 2018b)."

Critique 4. *While we assume that the center of mass for individual chromosomes and for the "chromosomal mass" (which we assume refers to the entire genome) is based on density rather than volume, it will be important to clarify this in the Results and Material and Methods.*

Response:
We would like to thank the review for this helpful comment. We acknowledge that "chromosomal mass" infers center of mass based on density, and have removed the term and replaced it with "3D DNA volume" or "DAPI stained chromosomes". The center of mass for a homolog or DNA was previously determined from the volume of a 3D reconstructed surface.

Critique 5. *We recommend that the information about the "boundary zone" as presented in Supplemental Figure 1 be included in the main text. Not only will readers have a better understanding of how the axes were drawn, the authors will have an opportunity to resolve concerns as to whether the discounting of chromosomes (e.g. 6/66 cells in the case of chromosome 1) could have affected outcomes.*

Response:
We thank the reviewer for this helpful suggestion. We agree that this information is important for understanding how chromosome positions were assigned and why a small number of cells were excluded from the positional analysis. We have moved this information from Supplemental Fig. 1 into the main Results section. This revision clarifies how the metaphase axis was defined and explicitly reports the number of cells excluded because homologous chromosomes were located within the boundary region.

The revised text now reads: "*For WCP experiments, the centrosomes were not able to be visualized. Therefore, a DNA-derived axis perpendicular to the longest axis of the 3D metaphase DNA plate was used to determine the centrosome axis. Immunofluorescence staining for γ-tubulin, along with a DNA counterstain, revealed that the centrosome axis and DNA-derived axis had a difference of 0.5 μM (Supplemental Fig. 1). Consequently, individual chromosome positions within ± 0.5 μM of the DNA center of volume, referred to as the "boundary region", were excluded from the analysis. For instance, out of 66 total cells, 6 cells exhibited homologous chromosome 1 in this boundary region, rendering the data uninterpretable (n=6/66 cells for chromosome 1).*"

© 2026. Published by The Company of Biologists under the terms of the Creative Commons Attribution License (https://creativecommons.org/licenses/by/4.0/).

Critique 6. *So that readers can better understand the breadth of sizes and configurations for homolog pairs, we suggest the authors select one chromosome and then show all the HUVEC and HAEC segmented images one cell at a time (e.g., in the spirit of Supplemental Figure 2 a'''). Such a montage would be quite compelling in terms of the authors' goals.*

Response:
We thank the reviewer for this great suggestion. We agree that presenting a montage would highlight the range of homolog sizes, orientations, and 3D spatial arrangements observed across individual cells and provides readers with a more comprehensive visual representation of the data. We have added a new Supplemental Figure 3, which presents 3D images of chromosome 19 from individual metaphase cells. Supplemental Figure 3 shows representative chromosome 19 configurations in HUVECs and HAECs.

Critique 7. *The authors say they chose to study chromosomes 17 and 19 because of their differences in basepair number, but other pairs of chromosomes (even if selected just from the pool of smaller chromosomes) show a much greater difference in size. While becomes clear later in the narrative that the authors wanted to avoid chromosomes carrying rDNA for the first part of their narrative, it remains unclear why they did not consider, for example, chromosomes 16, 18, or 20. Perhaps the authors could provide more detail for their reasoning.*

Response:
We thank the reviewer for this thoughtful suggestion. Our rationale for selecting chromosomes 17 and 19 was based on several considerations beyond chromosome size. We chose these chromosomes because they are both relatively small, non-acrocentric autosomes, allowing us to avoid potential complications associated with rDNA-containing acrocentric chromosomes.

In addition, chromosome 19 was of particular interest because it is one of the most gene-dense human chromosomes and has previously been reported to exhibit abnormal homologous chromosome pairing in both Caki-1 renal carcinoma cells and renal oncocytoma, where abnormal pairing of chromosome 19p has been associated with altered gene expression (Hua and Mikawa 2018; Koeman et al. 2008). These previous findings made chromosome 19 a biologically relevant candidate for investigating homologous chromosome organization in endothelial cells.

To clarify this rationale, we have added the following text: "*In addition, chromosome 19 is a gene-dense chromosome that has previously been reported to exhibit abnormal homologous chromosome pairing in Caki-1 renal carcinoma cells and renal oncocytoma (Hua and Mikawa 2018; Koeman et al. 2008).*"

We agree that chromosomes 16, 18, and 20 would also be valuable to examine. However, expanding the analysis to additional chromosomes is beyond the scope of the current study, and we plan to investigate these chromosomes in future work.

Critique 8. *We are curious why the authors did not study chromosome 14, as it also carries rDNA. (We do not at all mean to suggest that chromosome 14 needs to be included in the story.)*

Response:
We agree that chromosome 14 would be an interesting afrocentric chromosome to examine. However, it was not feasible for us to analyze all 23 chromosome pairs in the present study. Instead, we selected a representative set of ten chromosomes, including both autosomes and sex chromosomes, spanning a range of chromosome sizes and structural features, to provide a broad assessment of homologous chromosome organization. We have noted this as an interesting direction for future studies.

Critique 9. *We appreciate the authors providing average distances and average angular orientations, but including this information in the legends makes it difficult to compare these values across all experiments. Might the authors consider putting all of this information in one or two tables?*

Response:
We thank the reviewer for this helpful suggestion. We agree that consolidating these measurements into summary tables improves the accessibility of the data and facilitates comparisons across chromosomes and experiments. In response, we have added Supplemental Tables 3 and 4, which compile the average homologous chromosome distances and average

© 2026. Published by The Company of Biologists under the terms of the Creative Commons Attribution License (https://creativecommons.org/licenses/by/4.0/).

angular orientations for all experiments.

Critique 10. *The Materials and Methods indicate that HUVECs were commercially obtained in two samples, each representing 10 individuals, while the Results points out that experiments using HUVECs used a pool of 20 individuals. We suggest the authors clarify in the Results whether the two samples were pooled prior to culturing and, if not, which data were obtained from which sample. It would also be important to mention in Results that HUVECs were commercially obtained.*

Response:
We agree that the source and composition of the HUVEC samples should be described more clearly in the Results section. In response, we have revised the text to explicitly state that the HUVECs were commercially obtained and that the experiments were performed using two commercially available pooled HUVEC preparations, each representing 10 neonatal donors.

The revised text now reads: "*ImmunoFISH/WCP was carried out for samples of commercially available neonatal HUVECs (n=2 vials with 10 individuals pooled represented per vial, Supplemental Table 1).*"

Critique 11. *The data for HAECs are derived from 6 donors, but it is not clear in Figures 1 - 4 which data came from which donor. While some information could be extracted from Figure 5, it is time-consuming to do so, with outcomes being potentially ambiguous. Furthermore, due to the extensive use of color coding, the information in Figure 5 will not be fully accessible to readers who are color-blind. In short, we recommend the authors include this information in additional table(s). We also recommend that Figure 5b be annotated along the x axis so that chromosome and patient identity are unambiguous, with tick marks placed even when the pairing frequency is zero.*

Response:
We agree that the donor-specific data should be presented more clearly and that the organization of Fig. 5b could be improved to enhance readability and accessibility. We have added Supplemental Tables 2–4, which summarize the data for each individual HAEC donor and clearly indicate which donors were analyzed for each chromosome.

In addition, we have revised Fig. 5b to improve clarity by annotating the x-axis with the corresponding chromosome and donor identities. Tick marks are now included for all chromosome donor combinations, and the donor groups are clearly delineated.

Critique 12. *Would it be possible to have the authors comment on why the number of samples per chromosome is quite different between HUVECs and HAECs? For instance, for chromosome 17, the cell counts are 12 versus 82 for HUVECs and HAECs, respectively.*

Response:
The number of metaphase cells analyzed differs between HUVECs and HAECs because our primary objective in the present study was to characterize homologous chromosome organization in adult HAECs. HUVECs served primarily as a reference cell population, as we have previously demonstrated homolog segregation in neonatal HUVECs (Hua and Mikawa 2018, Cai, Casas, Quintero Plancarte, 2025). We performed additional HUVEC experiments to confirm the reproducibility of our previous findings, while focusing the majority of our data collection on the novel HAEC analyses.

In addition, HAEC cultures were technically more challenging to establish and maintain than HUVECs, making the acquisition of metaphase cells more labor-intensive for the MS graduate student co-authors. Despite these challenges, we prioritized collecting a larger HAEC dataset because it represents the central focus and novel contribution of the present study.

Critique 13. *We assumed that the differences in Figure 5b between HAEC samples are due largely to a stochasticity inherent in the system but, in light of all adult donors being associated with hypertension (was this true of all adult donors?), we are curious about other health-related characteristics of the donors. Thus, we suggest that all potential relevant health-related information be included in Results at the time that HAECs are first mentioned (or, if there is no additional information to make this clear). Here, too, we recommend that the commercial source for HAECs be mentioned in the Results.*

© 2026. Published by The Company of Biologists under the terms of the Creative Commons Attribution License (https://creativecommons.org/licenses/by/4.0/).

Response:
We agree that providing additional donor information improves the interpretation of the HAEC data. We have revised the Results section to describe the source of the HAECs, the available donor demographics, and the reported clinical information at the time the HAEC samples are first introduced. We also clarify that the HAECs were commercially obtained from Lonza and that hypertension was not reported for all donors.

The revised text now reads: "*HAECs were sourced from individual deceased organ donor patients of various ages, and purchased commercially (Lonza). Some donors had pathologies such as hypertension (n=2/7 patients). For example, one of the 50-year-old females reported hypertension and glioblastoma, while the other 50-year old-female reported fibromyalgia and acute alcohol-related pancreatitis. The 68-year-old male reported hypertension, hyperlipidemia, and diabetes. The other four remaining patients did not report pathologies. In addition, not all patients were analyzed for each chromosome.*"

Critique 14. *The authors mention the potential of aortic cells to be aneuploid. Did they encounter aneuploidy in their samples and, if so, how were such instances handled in terms of data acquisition and quantification?*

Response:
We did observe a small number of polyploid cells in our HAEC cultures. However, these cells were not included in the quantitative analyses, which were restricted to metaphase cells with the expected chromosome ploidy (2n) for the chromosome being examined. To clarify this point, we have added the following text to the Materials and Methods section: *"We detected a small population of HAECs to be polyploid, consistent with prior reports (Aviv et al. 2001; Borradaile and Pickering 2010). For example, we found 36 of approximately 207,000 HAECs to be polyploid, and these cells were not included in the analysis."*

Critique 15. *The Introduction does not provide sufficient background for haploid sets and antipairing. This is especially important because most readers will not be well-informed about these topics. Where else have haploid sets and antipairing been observed and, as importantly, where they have not been observed? The more readers know prior to reading the Results, the more easily they will recognize the advance of the authors over prior work. Thus, we recommend the authors move background information about haploid sets and antipairing from the Discussion ("Segregation of haploid genomes, or antipairing, has also been observed...to maintain overall genomic stability.") to the Introduction as well as provide considerably more detail. That haploid set organization defines the earliest stages of development (Reichmann et al. 2018, Mayer et al. 2000a,b,) makes it even more impactful that the authors observe a degradation of this pattern postnatally. The earlier readers are aware of this, the more exciting the Results will be. Similarly, knowing early on in the manuscript that antipairing may be an important process in Drosophila (Joyce, et al. 2012) will emphasize how the authors' findings likely extend across species - this is exciting! In brief, we urge the authors make better use of the Introduction to set the stage for their story. If word limits are an issue, we would recommend the authors shorten the more general introduction to 3D genome organization.*
Additional references to be included are:
Nagele et al. 1995 (PMID: 8525379) with respect to haploid sets
Nagele et al. 1998 (PMID: 9880766) with respect to haploid sets
Joyce et al. 2016 (PMC4939289) with respect to antipairing
Stamatov et al. 2025 (PMC11991918), a recent example where haploid sets and antipairing are not observed.

Response:
We thank the reviewer for this thoughtful and enthusiastic suggestion, as well as for highlighting several important references that strengthen the context of our study. We agree that the original Introduction did not provide sufficient background on haploid genome organization and antipairing for readers who may be unfamiliar with these concepts. We substantially revised the Introduction by moving and expanding material that was previously presented in the Discussion. We also incorporated the recommended references, including Nagele et al. (1995, 1998), Joyce et al. (2016), and Stamatov et al. (2025), to provide a more comprehensive overview of the current understanding of haploid genome organization and antipairing across model systems. The revised

© 2026. Published by The Company of Biologists under the terms of the Creative Commons Attribution License (https://creativecommons.org/licenses/by/4.0/).

Introduction now:

1. Defines haploid genome organization and antipairing and its significance during early development
2. Summarizes evidence for parental genome segregation in multiple model organisms, including mouse, bovine, rabbit, fruit fly, and the nematode
3. Introduces studies demonstrating that antipairing is an actively regulated process in *Drosophila*, while clearly distinguishing the *Drosophila* and mammalian systems
4. Includes studies in which haploid genome organization is not maintained
5. Introduces the concept that loss of haploid genome organization during aging may have important implications for genome organization and endothelial cell function

The revised Introduction now includes the following expanded background: "*Segregation of haploid genomes has also been observed in other model systems including the mouse, bovine, rabbit, fruit fly, and nematode (Aguilera et al. 2020; Hua et al. 2022; Keneklis and Odartchenko 1974; Maddox et al. 2005; Mayer et al. 2000a, b; Reichmann et al. 2018; Schneider et al. 2021; Joyce et al. 2012; Joyce et al. 2016; Gondos et al. 1970). This haploid set organization defines the earliest stages of development, with the observation of parental genome separation in mammalian models (Schneider et al. 2021; Reichmann et al. 2018, Mayer et al. 2000a, b). For example, in mouse and bovine cells, dual mitotic spindles at fertilization separate the two parental sets (Reichmann et al. 2018; Schneider et al. 2021). In addition, 5-bromodeoxyuridine (BrdU)-labeled sperm show paternal genome separation in mouse zygotes which persist until the four-cell stage (Mayer et al. 2000b). Electron microscopy of fertilized rabbit ova shows intact parental pronuclei adjacent to each other until approximately 21 hours after mating (Gondos and Bhiraleus 1970). After fertilization in the fruit fly, gonomeric cell divisions show the maternal and paternal genomes in apposition and structurally separate (Callaini and Riparbelli 1996; Loppin et al. 2015). These findings suggest a conserved mechanism. An evolutionary persistence may reflect selective pressure to limit harmful recombination and promote nuclear compartmentalization. In this study, we observe a degradation of the haploid set pattern postnatally.*

Antipairing may be an important process in the fruit fly, as it may be inhibited or enhanced by different regulators, suggesting there are mechanisms driving the antipairing of homologous chromosomes in different animal models (Joyce et al. 2012; Joyce et al. 2016). For example, in the early fruit fly embryo, a high-throughput FISH-based screen identifies genes involved in promoting or antagonizing somatic homolog pairing (Joyce et al. 2012). This finding suggests that there may be active antipairing mechanisms preventing the pairing of chromosomes in mammals. Taken together, these findings support the concept that antipairing is a biologically significant nuclear architecture strategy to maintain overall genomic stability.

Changes in chromatin structure further alter gene expression and increase susceptibility to DNA damage, with aging associated with higher levels of damage due to oxidative stress (Burgess et al. 2012; Wood et al. 2010; Wood and Helfand 2013; Zhang et al. 2023). Notably, the spatial arrangement of chromosomes within the cell may become disorganized in aged ECs, which can impact gene expression and overall cellular function (Ghosh and Meyer 2021; Heard and Bickmore 2007; Hua et al. 2022; Koeman et al. 2018; Lima et al. 2022; Misteli 2007). Aging disrupts nuclear organization through the loss of heterochromatin and nuclear lamina integrity, causing gene-rich regions to shift away from transcriptionally active domains, and altering gene expression patterns (Chandra and Kirschner 2016; Emerson and Lee 2023; López-Otín et al. 2013)."

Critique 16. *We believe the authors may have intended to cite Keneklis and Odartchenko 1973 (PMID: 4693953) rather than the 1974 paper by the same authors, as the latter is primarily a "control" for the former. Citing both references would also be fine.*

Response:
We thank the reviewer for carefully identifying this missing citation. We have added the 1973 reference and now cite both Keneklis and Odartchenko (1973) and Keneklis and Odartchenko (1974) in the Introduction where this work is discussed.

Critique 17. *"Loss of spatial segregation" and "loss of antipairing" could mean a complete absence of these processes, which we believe is a more extreme interpretation than intended. Perhaps the authors would consider alternative phrasing.*

© 2026. Published by The Company of Biologists under the terms of the Creative Commons Attribution License (https://creativecommons.org/licenses/by/4.0/).

Response:
We appreciate the concern that the phrases "loss of spatial segregation" and "loss of antipairing" could be interpreted as implying the complete absence of these processes. However, this is not our intended meaning.
See new text in Materials and Methods: “*The loss of spatial segregation was used to describe the position of individual homologs across the centrosome axis. The loss of antipairing was used to describe the loss of haploid set organization. Throughout the manuscript, we used these terms to describe the loss of statistically significant non-random spatial segregation, rather than the complete elimination of homolog separation. Our definition was based on statistical testing against the null hypothesis that homologous chromosomes were positioned randomly with respect to one another, similar to nonhomologous chromosome pairs (Hua and Mikawa, 2018).*”

Critique 18. *The proposal of a potential age-related impact on antipairing is interesting but, owing to the small sample size of adult donors (6) and undersampling of neonatal material in terms of fetal age and tissue type, we encourage the authors to be more conservative in their interpretations. Phrases that are problematic include (but are not limited to): "may decline with aortic endothelial cell age, leading to increased susceptibility to abnormal pairing and cardiovascular disease (Abstract, page 3,)", "trend of increased susceptibility of pairing throughout age (subtitle and figure legend, pages 22 and 25, respectively)", and "after birth, there may be an increased susceptibility (narrative, page 22)". A more conservative tone can be achieved through rewording statements, reminding readers of the small sample sizes and thus tentativeness of the proposals, and moving statements to the Discussion. These changes would be in line with the authors' acknowledgment of limitations in the Discussion and proposal of the kinds of concrete future experiments that are needed.*

Response:
We thank the reviewer for this constructive suggestion. We agree that, given the limited number of adult donors and the differences between the neonatal and adult endothelial cell populations, our conclusions regarding age-related changes should be presented more cautiously. We have revised the manuscript throughout to adopt a more conservative interpretation of the data. Specifically, we have softened statements in the Abstract, Results, and Discussion that could be interpreted as implying a definitive age-dependent effect.

We now emphasize that our observations are preliminary, acknowledge the relatively small donor cohort, and explicitly state that larger studies across a broader range of ages will be required to determine whether the observed changes are associated with aging. In addition, we have moved speculative statements from the Results to the Discussion, where they are presented in the context of the study's limitations and future directions.

Critique 19. *Similarly, the authors discuss a potential correlation between the reduced frequency of antipairing and cardiovascular disease but do not provide functional evidence connecting the two. This angle would benefit from more cautious framing.*

Response:
We agree that our study does not provide functional evidence linking reduced antipairing with cardiovascular disease. To avoid overstating the implications of our findings, we have removed language suggesting this potential association from the manuscript.

Critique 20. *As the definition of "pairing" is fairly stringent and rigid, we wonder whether such strictness could have led the authors to miss subtle patterns. In other words, would a window of distance/angle thresholds (rather than a simple cut-off) capture a gradient of pairing disruption and, from there, additional discoveries? Although we are curious whether this approach will allow the authors to glean additional insights, we are not requiring that this approach be taken.*

Response:
We agree that evaluating homolog pairing as a continuum could provide additional biological insights in future studies. However, in the present study, our definition of homolog pairing was based on experimentally defined imaging and analytical criteria rather than an arbitrary threshold. The minimum distance threshold is constrained by the spatial resolution of our confocal

© 2026. Published by The Company of Biologists under the terms of the Creative Commons Attribution License (https://creativecommons.org/licenses/by/4.0/).

imaging system, such that a measured distance of 0 µm corresponds to homologs that are within the optical resolution limit (~ 300 nm) and are therefore interpreted as being in direct spatial juxtaposition. The angular measurements were calculated using our previously established 3D vector analysis, which is described in greater detail in the revised Materials and Methods. These criteria were selected to provide a consistent and reproducible definition of homolog pairing across all samples.

We have expanded the Materials and Methods section to more fully describe the distance and angular measurements, the rationale for the thresholds used, and the analytical approach for quantifying homolog pairing.
See text: "*Homologous chromosomes were identified as paired if the distance between them was 0 µm (≤0.36 µm, the spatial xy resolution limit of the confocal microscope) and an angular orientation (vector angle) of 0°.*

Minimal distances between homologs were determined by the shortest distances between the edges of the 3D surfaces of homologs.

The 3D vector coordinates determined the angular orientation of the homologs. To determine a 3D vector coordinate, a 3D surface of a homolog was selected. The longest ellipsoid axis length (Ellipsoid Axis C) was identified in the "specific values" tab. The x, y, and z coordinates were then acquired to define the 3D vector. This process was repeated for the other homolog. The vector angle was calculated by taking the inverse cosine of the normalized dot product."

While we agree that analyses based on graded distance or angular thresholds may reveal additional patterns of chromosome organization, such analyses are beyond the scope of the present study and represent an interesting direction for future work.

Critique 21. *Do the authors have direct (or even indirect) evidence confirming that their samples represent normal karyotypes? Along these lines, did the authors come across situations where only one homolog was visible? If so, how did the authors distinguish monosomy from an instance of pairing that was extensive enough to preclude the distinction of two homologs?*

Response:
We thank the reviewer for this important question. Although we did not perform conventional karyotyping on each donor sample, several lines of evidence support that the cells analyzed represented predominantly normal karyotypes. First, all HUVEC and HAEC cultures were obtained from commercially validated sources with quality control performed by the vendor (ATCC and Lonza). Second, the vast majority of metaphase cells exhibited the expected number of chromosome paint signals for the chromosomes analyzed.

We also expanded the Materials and Methods section to describe the use of chromosome paint signal volume analysis to identify paired homologs and to distinguish these events from potential monosomy.

With respect to homolog pairing, when homologous chromosomes were paired, they appeared as a single chromosome paint signal rather than two spatially separated signals.

See text in Materials and Methods: "*To distinguish homolog pairing from chromosome loss (monosomy), we quantified chromosome paint signal volume. The volume of a paired chromosome paint signal was approximately equivalent to the combined volume of two individual homologs. For example, the average volume of an individual, antipaired, chromosome 19 homolog paint signal was measured to be 2.15 ± 1.02 µm^3 SD in 10 HUVECs and 2.32 ±1.08 µm^3 SD in 10 HAECs. However, the average volume of the single chromosome paint signals, in the four cases of chromosome 19 pairing in HAECs, was 5.12 ± 3.38 µm^3 SD. These average volumes showed that the paired chromosome 19 paint signals in HAECs were approximately twice the volume of the individual, antipaired, homologous chromosome 19 paint signals in HUVECs and HAECs respectively.*"

This volume analysis suggests that the single chromosome paint signal in HAECs is representative of two homologous chromosomes that are paired.

Critique 22. *We are curious whether the authors took other features of the homologs into account (e.g., chromosome shape and size) and, if so, were there any correlations between these features and antipairing?*

© 2026. Published by The Company of Biologists under the terms of the Creative Commons Attribution License (https://creativecommons.org/licenses/by/4.0/).

Response:
We did consider chromosome size and morphology in our analyses. One of the main findings of this study is that chromosome-specific differences exist in the maintenance of homolog spatial segregation. We observed that several of the smaller autosomes exhibited a greater loss of spatial segregation and a higher frequency of abnormal homolog pairing than larger autosomes and the X chromosomes (see Fig. 5 and Supplemental Table 2).

These observations suggest that chromosome size and/or structural characteristics may influence the maintenance of homolog antipairing during aging. However, because only a subset of chromosomes was analyzed, we cannot determine whether chromosome size alone accounts for these differences. Other chromosome-specific features, such as gene density, chromatin organization, nuclear positioning, or sequence composition, may also contribute.

We have also expanded the Discussion to note the chromosome-specific differences observed in our study while avoiding conclusions that extend beyond the current dataset. We also refer readers to Fig. 5 and Supplemental Table 2, which summarize the relative frequencies of loss of spatial segregation and abnormal homolog pairing across the chromosomes examined.

Critique 23. *We discovered several discrepancies within figures, between figures, and between the text and figures (see below) and, as we only spot checked the manuscript, there may be other discrepancies in the manuscript. We urge the authors to doublecheck all their data and calculations, including their statistical tests. Examples of what we noticed:*
a) Figure 1n reports 83 cells, while Figure 5a reports only 82.
b) Figure 1s indicates a total of 99 cells for chromosome 17, while Figure 5a notes 94. As would be expected, the two P values for these figures also differ.
c) Figure 2y indicates a 0.29 pairing frequency for chromosome 15 in HAECs, while Figure 2r would indicate a frequency of 0.28 (14/50).
d) On page 18 (lines 44-50), the authors compare the pairing frequency of the larger chromosomes (1, 4) with that of the acrocentric chromosomes (13, 15, 21, 22), citing a total of 320 cells. However, Figure 2y reports a total of only 200 HAEC cells for acrocentric chromosomes. We believe the total of 320 includes the data for non-acrocentric small chromosomes 17 and 19. As such, the frequency of pairing, will change, as will the outcome of the chi-square test.
e) On page 18 (lines 50-55), the authors report a pairing frequency of 14.18% for chromosomes 13, 15, 17, 19, 21, and 22 (61/430), but we believe 430 should be replaced with 320, as 430 likely includes the cell counts for chromosomes 1 and 4 as well. Furthermore, we feel the count of 61 overstates the number of paired homologs. (Note, if the total HAECs with respect to chromosome 17 is actually 83 instead of 82, then the correct total should be increased by 1 to 321.)
f) The counts for XY HAEC cells in Figures 4l and 4m are 44, while Figure 5a reports 43.

Response:
We thank the reviewer for the careful examination of the manuscript and for identifying these discrepancies. We appreciate the time taken to cross-check the figures, text, and calculations! We thoroughly re-examined the entire dataset, recalculated all pairing frequencies and statistical analyses, and carefully verified that the cell counts are consistent throughout the manuscript, figures, figure legends, and supplemental materials.

We corrected the inconsistencies in cell counts and pairing frequencies identified by the reviewer, updated the corresponding statistical analyses where necessary, and revised the text to clearly indicate which chromosome groups and donor samples were included in each comparison. We also added Supplemental Tables 2–4 to provide a comprehensive summary of the underlying data, allowing readers to readily verify the cell counts and quantitative measurements used throughout the study.

Minor points:

1) We encourage the authors to increase the font size for Figures 1a and 1s and for Figure 2, throughout. Please also increase the size of the red P in Figures 1- 4.

Response:
We have increased the font size in Figures 1A, 1S, and all panels of Fig. 2. We have also increased the size of the red "P" labels throughout Figs. 1–4 to improve visibility.

© 2026. Published by The Company of Biologists under the terms of the Creative Commons Attribution License (https://creativecommons.org/licenses/by/4.0/).

2) Figure 1b: Would it be possible to reorder the nuclei to match the order of the data in the graph below?

Response:
We have revised the schematic in Fig. 1B by reordering the nuclei to match the order of the corresponding data presented in the graph below.

3) Figures 1d and 1e: The color scheme and how homologs were designated one color or the other is not immediately clear. One solution would be to move Supplementary Figure 2 into the main text.

Response:
We have expanded the Materials and Methods section to clarify how homologs were identified, color-coded, and analyzed. We now explain that each homolog was analyzed individually based on its 3D center of volume, which was used for all positional, distance, and angular measurements. We also added additional details describing how the center of volume for each homolog was determined during the image analysis. We also moved Supplemental Figure 2 into the main text as suggested to provide an explanation of the color scheme and chromosome segmentation used.

4) We suggest Fig. 1s conform to the style of Fig. 2y, 3m, and 4m, enumerating sample size for each assay.

Response:
We have revised Fig. 1S to conform to the style used in Figs. 2Y, 3M, and 4M by including the sample size (n) for each.

5) We recommend that the number of paired homologs be given above the bars in Figures 1s, 2y, 3m, 4m, 5a, and 5b, as the y-axis is already providing pairing frequencies. We suggest this because we often found ourselves back calculating the number of paired homologs.

Response:

We have added the number of paired homologs above the bars in Figs. 1S, 2Y, 3M, 4M, 5A, and 5B.

6) The data points are too difficult to distinguish in Figures 1n, 2x, 4i. One solution would be to put some of the cell numbers outside the mass of datapoints, connecting them to their nucleus with lines.

Response:
We explored this approach by placing some of the cell numbers outside the group of data points and connecting them to the corresponding dot with lines. However, because of the density of the data, this resulted in a cluttered figure that reduced overall readability. We therefore decided to retain the original presentation, as we believe it provides the clearest visualization of the data.

7) Figure 5b: We suggest the authors remove "Groups" from title because this term could imply that there were multiple donors for the younger and older ages.

Response:
We have removed the term "Groups" from the title to avoid implying that multiple donors were represented within each age category.

8) Supplemental Table 1: This table can be simplified by removing "n=" and "cells" wherever possible.

Response:
We have revised Supplemental Table 1 by removing "n =" and “cells".

<u>Second decision letter</u>

© 2026. Published by The Company of Biologists under the terms of the Creative Commons Attribution License (https://creativecommons.org/licenses/by/4.0/).

MS ID#: jcs.264668R1

MS TITLE: Higher frequency of homologous chromosome pairing in adult endothelial cells as compared to neonatal endothelial cells

AUTHORS: Jemery Morales; Omar Akhtar; Gabriel Quintero Plancarte; Lisa L Hua

ARTICLE TYPE: Research Article

Dear Dr Hua,

Thank you for submitting your revised manuscript to JCS for further consideration. As you will see, the reviewers appreciated how you and your group have addressed their critiques and now enthusiastically support publication. I am returning the manuscript to you as a "minor revision" simply to give you the opportunity to address a few minor suggestions for additional changes to the text from the reviewers. I hope that you will be able to address these small points quickly because I would like to be able to accept your paper so that we can move forward with publication as soon as possible.

Reviewer 1

SUMMARY OF THE ADVANCE MADE IN THIS PAPER AND ITS POTENTIAL SIGNIFICANCE TO THE FIELD

This manuscript makes important contributions to our understanding of chromosome organization in cells from human neonatal and adult endothelial tissues. The authors carefully and comprehensively document segregation of parental chromosome sets in the two cells types and uncover interesting chromosome group-effects (e.g. based on size, sex chromosomes, acrocentric or nonacrocentric). The introduction and discussion place the results in contexts of what is known about the process in different species.

SUGGESTIONS TO AUTHORS

The authors have addressed my major comments. The paper is much easier to read with the new organization of the discussion and introduction sections. The description of donors is helpful.

Minor comments
I have only two minor comments. There are several instances where the authors state they "previously demonstrated" some effect. Since the work is published and is cited, the commentary on previous work is not necessary. The other is that there is still an extended description of future experiments in the discussion section that detracts from the central findings. Both of these are stylistic issues that the authors can choose to incorporate or not.

Reviewer 2

SUMMARY OF THE ADVANCE MADE IN THIS PAPER AND ITS POTENTIAL SIGNIFICANCE TO THE FIELD

SUGGESTIONS TO AUTHORS

The authors have addressed our concerns, and we are thrilled to see the revised manuscript. We have only two comments at this point:

Regarding Critique 4, may we suggest the following wording (Line 276, add "segmented"): The position of individual chromosomes was determined by identifying the center of volume of the segmented chromosome paint signal (Fig. S2A).

Regarding Critique 15: The expanded background will greatly help readers understand the implications of their study. However, might the authors consider adding one additional layer of clarification to the following three statements? These additional edits will help the reader better place the authors' studies in the context of the entire field.

© 2026. Published by The Company of Biologists under the terms of the Creative Commons Attribution License (https://creativecommons.org/licenses/by/4.0/).

a) Line 148: "In this study, we observe a degradation of the haploid set pattern postnatally." Please clarify whether this statement pertains to interphase or mitotic cells and somatic or germline cells.

b) Line 153: "For example, in the early fruit fly embryo, a high-throughput FISH-based screen identifies genes involved in promoting or antagonizing somatic homolog pairing." Please clarify whether this applies to interphase or mitotic cells.

c) Line 162: "Notably, the spatial arrangement of chromosomes within the cell may become disorganized in aged ECs, which can impact gene expression and overall cellular function." Please clarify whether this refers to interphase or mitotic cells.

Second revision

Author response to reviewers' comments

Please find our revised manuscript attached (#jcs.264668R1). We would like to thank both reviewers for their helpful comments and constructive criticisms to improve our manuscript. We have incorporated their final suggestions and appreciate their feedback. Our point-by-point responses to each of the reviewer's comments are provided below.

Reviewer #1
Minor comments

Critique #1: *There are several instances where the authors state they "previously demonstrated" some effect. Since the work is published and is cited, the commentary on previous work is not necessary.*

Response:
We appreciate the Reviewer's careful reading of the manuscript and agree that these statements are unnecessary given that the prior work has been published and cited. Accordingly, we have removed instances of "previously demonstrated" and similar language from the main text including the abstract, results, discussion, and materials and methods.

Critique #2: *The other is that there is still an extended description of future experiments in the discussion section that detracts from the central findings. Both of these are stylistic issues that the authors can choose to incorporate or not.*

Response:
We appreciate the Reviewer's thoughtful suggestion. To maintain focus on the central findings of the study, we have removed the extended description of proposed future experiments from the Discussion.

Reviewer #2
Minor comments
Regarding Critique 4, *may we suggest the following wording (Line 276, add "segmented"): The position of individual chromosomes was determined by identifying the center of volume of the segmented chromosome paint signal (Fig. S2A).*

Response:
We appreciate the Reviewer's suggested wording and have incorporated "segmented" into the revised text as recommended (see revised text: "*The position of individual chromosomes was determined by identifying the center of volume of the segmented chromosome paint signal (Fig. S2A').")*
Regarding Critique 15: *The expanded background will greatly help readers understand the implications of their study. However, might the authors consider adding one additional layer of clarification to the following three statements? These additional edits will help the reader*

© 2026. Published by The Company of Biologists under the terms of the Creative Commons Attribution License (https://creativecommons.org/licenses/by/4.0/).

better place the authors' studies in the context of the entire field.
a) Line 148: "In this study, we observe a degradation of the haploid set pattern postnatally." Please clarify whether this statement pertains to interphase or mitotic cells and somatic or germline cells.
b) Line 153: "For example, in the early fruit fly embryo, a high-throughput FISH-based screen identifies genes involved in promoting or antagonizing somatic homolog pairing." Please clarify whether this applies to interphase or mitotic cells.
c) Line 162: "Notably, the spatial arrangement of chromosomes within the cell may become disorganized in aged ECs, which can impact gene expression and overall cellular function." Please clarify whether this refers to interphase or mitotic cells.

Response:
We appreciate the Reviewer's suggestion to provide additional clarification and context for these statements. We have revised the text throughout this section to specify that our findings pertain to mitotic somatic cells and have explicitly identified studies involving interphase cells where appropriate. These revisions more clearly distinguish between mitotic and interphase cells and place our findings within the broader context of the field. See revised text:

a) *"In this study, we observe a degradation of the haploid set pattern in somatic mitotic cells, postnatally."*
b) *"For example, in the early fruit fly embryo, a high-throughput FISH-based screen identifies genes involved in mitotic and interphase processes, promoting or antagonizing somatic homolog pairing (Joyce et al., 2012)."*
c) *"Notably, the spatial arrangement of chromosomes within the mitotic cell may become disorganized in aged ECs, which can impact gene expression and overall cellular function in interphase cells (Ghosh and Meyer, 2021; Heard and Bickmore, 2007; Hua et al., 2022; Koeman et al., 2008; Lima et al., 2022; Misteli, 2007)."*

Third decision letter

MS ID#: jcs.264668R2

MS Title: Higher frequency of homologous chromosome pairing in adult endothelial cells as compared to neonatal endothelial cells

Authors: Jemery Morales; Omar Akhtar; Gabriel Quintero Plancarte; Lisa L Hua

Article Type: Research Article

Dear Dr Hua,

I am happy to tell you that your manuscript has been accepted for publication in Journal of Cell Science, pending standard publication integrity checks.

© 2026. Published by The Company of Biologists under the terms of the Creative Commons Attribution License (https://creativecommons.org/licenses/by/4.0/).
